# Temporal dynamics of socioeconomic inequalities in depressive and anxiety symptoms during the COVID-19 pandemic: a scoping review

**DOI:** 10.3389/fpubh.2024.1397392

**Published:** 2024-07-03

**Authors:** Kiara Herrmann, Florian Beese, Lina Wollgast, Elvira Mauz, Christina Kersjes, Jens Hoebel, Benjamin Wachtler

**Affiliations:** ^1^Institute of Public Health, Charité – University Medicine Berlin, Berlin, Germany; ^2^Department of Epidemiology and Health Monitoring, Robert Koch Institute, Berlin, Germany

**Keywords:** mental health, depressive symptoms, anxiety symptoms, COVID-19 pandemic, socioeconomic inequalities

## Abstract

**Background:**

The existence of socioeconomic inequalities in the prevalence of symptoms of depression and anxiety is widely acknowledged, and individuals from lower socioeconomic backgrounds tend to exhibit higher rates of symptoms. However, the direction in which the COVID-19 pandemic has influenced these disparities remains uncertain. We therefore aimed to systematically outline the available evidence on the temporal dynamics of socioeconomic inequalities in symptoms related to depression and anxiety during the COVID-19 pandemic across high-income countries.

**Methods:**

A scoping review was conducted by searching the databases Embase, Scopus and PsycINFO. According to pre-defined eligibility criteria, two reviewers independently screened titles and abstracts as well as full texts of the compiled records. Data from the included studies were extracted using a standardised data-extraction form and analysed numerically and narratively. The scoping review followed the PRISMA-ScR guidelines.

**Results:**

A total of 49 studies comprising 149 analyses of socioeconomic indicators in relation to symptoms of depression and anxiety were included. Despite heterogeneous study designs and results, there was a tendency of increasing (40.9%; *n* = 61) or persistent (38.2%; *n* = 57) inequality trends to the detriment of those in socially more disadvantaged positions. Increasing inequalities were most pronounced when income was used as a socioeconomic indicator. Groups with lower socioeconomic status appeared most vulnerable in the initial phase of the COVID-19 pandemic. Throughout the pandemic, dynamics were diverse, with persistent trends most frequently reported.

**Conclusion:**

Overall, to the detriment of those with lower socioeconomic status, mental-health inequalities persisted or increased in most analyses. Continually monitoring socioeconomic inequalities over time is crucial, since this makes it possible to adapt prevention and intervention strategies to specific pandemic phases. Interventions targeting job security, income security and educational attainment could reduce mental-health inequalities. The results can contribute to preparedness plans for future pandemics and crises.

## Introduction

Socioeconomic inequalities in the prevalence of common mental-health disorders, such as depression and anxiety disorders, are well known, with individuals from more disadvantaged socioeconomic backgrounds having higher prevalence of symptoms (1, 2). Individuals suffering from material deprivation, unemployment, limited access to education and social isolation are particularly vulnerable to symptoms of depression and anxiety (3).

On March 11, 2020, the World Health Organization declared COVID-19 a pandemic (4). As one of the consequences, the COVID-19 Mental Disorders Collaborators predicted a global rise of 27.6% in cases of major depressive disorder and a 25.6% increase in anxiety disorder cases in 2020 (5). Reviews showed that symptoms of common mental disorders increased during the first period of the COVID-19 pandemic (6–8). A decline in symptoms was observed in the course of the pandemic, but figures did not return to pre-pandemic levels (7).

The increase in common mental-health disorders (5) was attributed to many changes in daily routines and the associated stress. Many people experienced resource depletion when being furloughed, dismissed or forced to work under new conditions (9, 10). In addition, policy measures were implemented to curb the spread of the virus, encompassing actions such as the closure of childcare facilities and educational institutions, disrupting routine family dynamics (11). In addition, face-to-face psychosocial services were severely compromised (12). It is plausible that the compounding stressors arising from the COVID-19 pandemic amplified pre-existing mental-health challenges (13). Investigations have indicated a notable global escalation in the occurrence of psychopathologies associated with common mental disorders since the onset of the pandemic (5, 12, 14, 15).

The stressors created by the COVID-19 pandemic may have affected certain socioeconomic groups more severely and may also have had different effects depending on socioeconomic status (SES) (16). As with health inequalities in general, socioeconomic inequalities in symptoms of depression and anxiety disorders may change or reproduce over time, leading to specific social-epidemiological patterns of disease distribution at different stages of the pandemic (17).

In the context of the possibly differential increase in symptoms of mental disorders depending on individuals’ socioeconomic status during the COVID-19 pandemic, the following two theories may serve as theoretical background. The conservation of resources (COR) theory (18) posits that individuals strive to amass and safeguard resources to shield themselves from adversity and manage the requisites of daily life. Resources include valuable circumstances or contexts, personal attributes like self-efficacy, and tangible assets like financial means. A fundamental tenet of this theory underscores that the loss of – or perceived threat to – resources can impact on well-being (19). Despite its apparent paradox, the COR theory acknowledges that individuals with more resources might encounter diminished well-being in specific contexts. The extent of well-being impairment hinges upon how much their personal resources contract within that context (20). In the context of the COVID-19 pandemic, individuals with a higher SES might have experienced a greater detrimental impact on mental health due to greater resource depletion (11).

The second theory is the vulnerability-stress model (21). This model delineates the interactions between vulnerability and stress. The central premise is that both elements are prerequisites for the onset of a mental disorder (21). Vulnerability factors encompass (neuro) biological, psychological and environmental elements of vulnerability (22), contributing to an individual’s susceptibility to mental disorders. The COVID-19 pandemic and its containment measures can be construed as stressors. Assuming heightened vulnerability among individuals with more disadvantaged SES (23), e.g., due to limited financial resources etc., experiences during the COVID-19 pandemic might have more frequently led to symptoms of common mental disorders (24).

First results from studies into changing socioeconomic inequalities in common mental disorders are inconclusive and sometimes contradictory. Some studies from the early stages of the COVID-19 pandemic suggested that socially disadvantaged groups were disproportionately affected by the COVID-19 pandemic and were at increased risk of developing common mental symptoms (13, 25, 26). However, contrasting findings from the USA suggest, for instance, that individuals with a higher education in the USA experienced a significant surge in depressive symptoms during the COVID-19 pandemic (11). Conversely, some studies indicate no differentiation in escalating mental-health problems and depressive symptoms across various SES groups (16, 27). However, comparisons were made between different periods, with some studies comparing to pre-pandemic levels (11, 16, 27) and some exclusively using peri-pandemic data (28–30). Understanding the time-dependent patterns of socioeconomic inequalities in mental-health complaints during the COVID-19 pandemic could help identify high-risk groups at different stages of the pandemic and enable timely public-health interventions to reduce mental-health inequalities and the overall burden of disease.

We therefore conducted a systematic scoping review to address the following research question: what is the evidence relating to trends in socioeconomic inequalities in symptoms of common mental-health disorders in high-income countries during the COVID-19 pandemic? To the best of our knowledge, this is the first systematic review of the evidence on SES-specific changes in symptoms of common mental disorders during the COVID-19 pandemic.

## Methods

We conducted a systematic scoping review by searching the databases Scopus, Embase and PsycINFO in order to identify, collate, map and finally synthesise the available evidence (31, 32). Scopus offered a comprehensive compilation of international medical and social scientific publications relevant to the research question (33). Embase covered biomedical journals and included Medline records from 2010 onwards (34), while PsycINFO complemented the database search with psychology-related publications (35). A detailed study protocol was published on the Open Science Framework (36) prior to the start of the study. This review follows the preferred reporting items for systematic reviews and meta-analyses extension for scoping reviews (PRISMA-ScR) guidelines (37).

### Search strategy

This review concentrated on four main concepts: (1) socioeconomic inequalities, (2) symptoms of common mental-health disorders, (3) COVID-19, and (4) longitudinal design. Due to comparability concerns (38, 39), low- and middle-income countries were excluded based on the World Bank’s list published in 2021 (40). Database-specific search strings were developed for each concept (see Supplementary material S2), tailored to the respective databases, including relevant expanding terms like Emtree thesaurus for the Embase search. A scientific librarian was consulted during the search string development process. An additional filter was used to restrict the publication time from 2020 until the day of the search in 2023. Search terms were applied to search within titles and abstracts. The search in the electronic databases Scopus, Embase and PsycINFO was conducted on 14 May 2023.

### Eligibility criteria

The review only considered literature meeting predefined eligibility criteria (Table 1).

**Table 1:** Eligibility criteria.

Category	Inclusion	Exclusion
Study design	Cohort studies with at least two measurements Repeated cross-sectional studies with at least two measurements	Studies that do not own data analysis Studies in which the measurements took place exclusively before the COVID-19 pandemic
Publication	Peer-reviewed articles reporting the results of original research	Study preprints Study protocols Comments, conference contributions and scientific communications Essays
Population	General population at the regional or national level Representative of the national or regional population Adults	Subgroups of population with certain conditions (e.g. outpatient clinic patients) The population is not studied at the regional or national level but at the level of a city or a neighbourhood Population studied: children and adolescents
Socioeconomic status	Indices of individual-level socioeconomic status Income Education Occupation, incl. Employment Area-level socioeconomic indices	Studies with an exclusive focus on race/ethnicity
Outcome	Psychopathological symptoms of depression Psychopathological symptoms of anxiety disorders Psychopathological symptoms of depression and anxiety combined Measures via any questionnaires (e.g. PHQ-9, PROMIS, Becks Depression-Inventory II) in any form (e.g., interview in person, interview by phone, written questionnaire)	Exclusive focus on suicide Other outcomes (e.g., well-being, life satisfaction, psychopathological symptoms in general, COVID-19 related anxiety, and stress)
Country	High-income countries (40)	Low−/lower-middle and upper-middle income countries (40)
Language	English and German	All other languages

The selected studies were required to examine temporal dynamics in symptoms of common mental disorders during the COVID-19 pandemic, i.e., measures against depression or anxiety associated with socioeconomic factors had to be assessed at least at two different time points. Depressive and anxiety disorders are known to be frequently underdiagnosed in healthcare settings, and diverse access barriers might lead to an undertreatment (41). We therefore exclusively included studies that systematically ascertained symptoms via primary data collection in the general population and studies that used only secondary data; e.g. settlement data from health insurances were excluded to reduce selection bias. The initial time points may have preceded the onset of the COVID-19 pandemic, while the subsequent time points can have been during the pandemic. Alternatively, data collection might have taken place at two time points during the COVID-19 pandemic. Hence, cohort studies and repeated cross-sectional studies were eligible for inclusion. In scoping reviews there is typically no assessment of the quality of evidence conducted due to the heterogeneity of the included studies (31, 32). Peer-reviewed studies are generally subject to a minimum standard level of quality control. In contrast, non-peer reviewed publications, such as study preprints, do not have such requirement and hence were excluded. The target population encompassed the general population at a national or at least regional level. Studies with relatively homogeneous study populations (e.g., specific occupational cohorts) were excluded, as they did not permit systematic comparisons of risks between socioeconomic groups and might be particularly prone to collider bias.

### Study selection and descriptive statistical analysis

Two reviewers (KH and LW) independently screened the titles and abstracts of the records obtained from the database search and the full texts of the identified studies. The software Rayyan (42) supported the title and abstract screening. To assess interrater reliability, Cohen’s Kappa coefficient was calculated for both stages of study selection (43). In cases of disagreement, the records were discussed until a consensus was reached. A PRISMA flow chart summarised and visually represented the selection process. Microsoft Excel was used for all descriptive statistical analyses and the calculation of the Cohen’s Kappa coefficient.

### Data charting process

A standardised data-extracting chart was designed in an iterative process. After agreement on the data chart was reached, the following information was extracted from each included full-text article: author (year), country, number of participants, study design, observation period, outcome, socioeconomic indicator, outcome measures (e.g., odds ratio), most-affected SES-group, inequality trend, absolute or relative outcome measure. The results of temporal dynamics in socioeconomic inequalities were categorised into four groups: (1) persistent, (2) increasing, (3) decreasing or (4) crossing over time. For example, increasing inequalities were assumed if the prevalence difference between the highest and lowest socioeconomic category increased over time. Socioeconomic indicators were categorised based on income, education, occupation (including employment) or index-based socioeconomic measures. In the following, the term study refers to the published study, including all analyses, and the term analysis refers to the one particular analysis that was reported by the study.

### Evidence synthesis

First, all the articles were summarised in a table based on these previously defined components and adjusted in an iterative process. This table provided an overview of the temporal patterns observed for each outcome and socioeconomic indicator. Second, the principal descriptive results were summarised graphically or numerically. Finally, these preliminary descriptive results were used to narratively synthesise the evidence (44).

## Results

**Figure 1:**
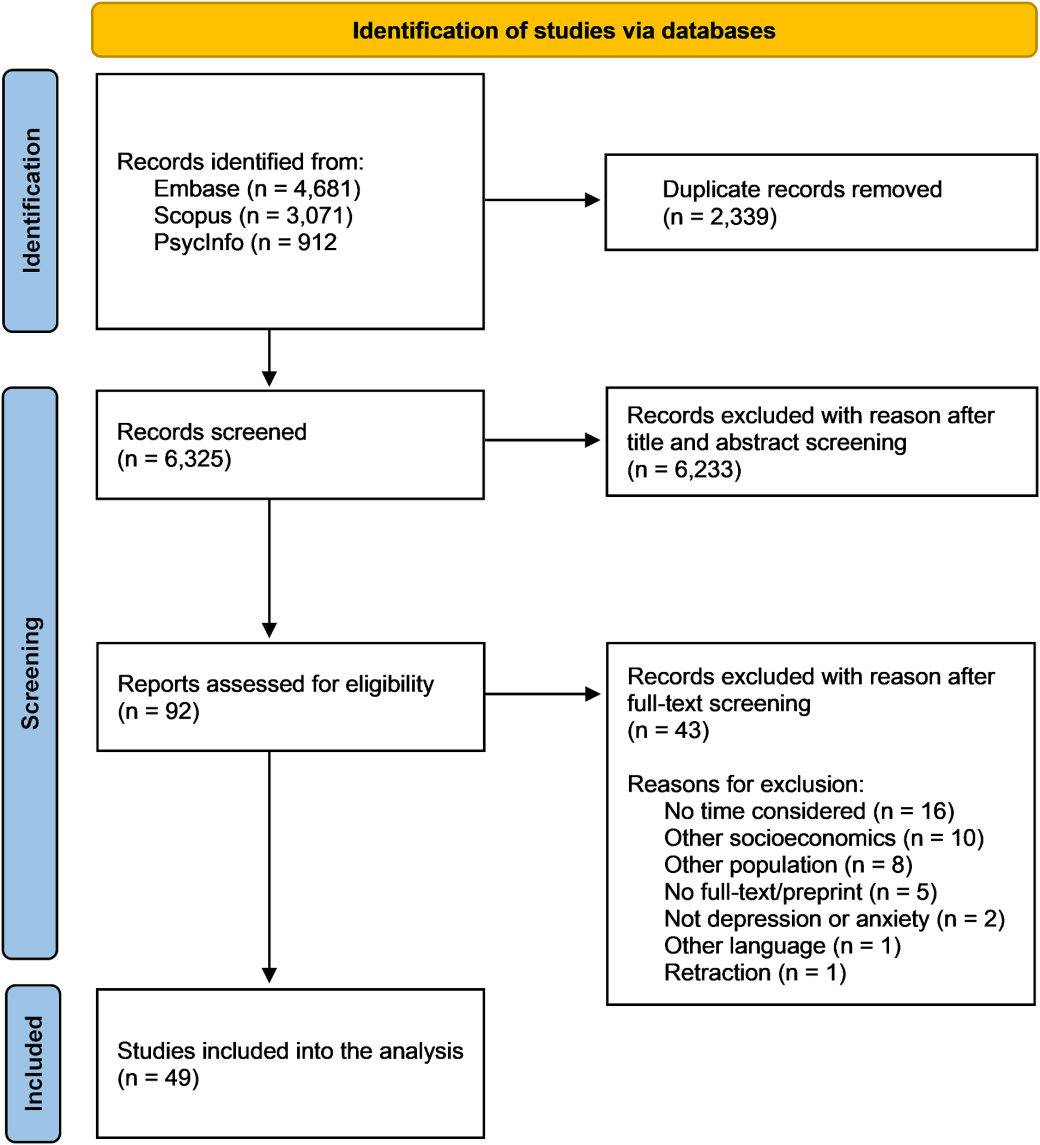
PRISMA flow chart of the study selection process.

Out of all 8,664 database records identified by our search, 49 articles met all the eligibility criteria comprising a total of 149 analyses that combined different socioeconomic indicators with symptoms of depression and anxiety over time. The study selection process is presented in detail in Figure 1.

The Cohen’s Kappa coefficient in the title and abstract screening was 0.931. The interrater percent agreement was 99.8%. In the full-text screening, Cohen’s Kappa coefficient was 0.799. The interrater percent agreement was 90.0%.

An overview of the included studies and their main findings is presented in Table 2.

**Table 2:** Summary table of the included studies and their main findings.

First author, year	Country	Number of participants	Study design	Observation period	Outcome	Soicoeconomic indicator	Outcome measure	Most affected SES-group	Inequality trend	Relative/ absolute measure-ment
Cha et al., 2022 (45)	KR	*n* = 444,051	Repeated cross-sectional	Aug 2019 – Okt 2020	Depression	Occupation	Odds ratio	Unoccupied	Persistent	R
						Education	Odds ratio	Lowest (male) and middle (female)	Persistent	R
						Income	Odds ratio	Lowest	Persistent	R
Coley and Baum, 2022 (46)	USA	*n* = 1,302,455	Repeated cross-sectional	Apr 2020 – Nov 2020	Depression	Education	Prediction rate, odds ratio	Lowest and second lowest	Decreasing	R
					Anxiety	Education	Predication rate, odds ratio	From lowest to highest	Crossover	R
Daly and Robinson, 2020 (47)	USA	*n* = 7,319	Cohort	Mar 2020 – Jul 2020	Depression & anxiety	Income	Regression estimates for the difference in increase and decrease, covariate adjusted (participant age, sex, race/ethnicity, household income, and the presence of a pre-existing mental health condition)	Lowest	Decreasing	R
Dragano et al., 2022 (48)	DE	*n* = 161,787	Cohort	2014 – Nov 2020	Depression	Occupation	Adjusted regression coefficient	Job insecurity high	Increasing	R
						Income	Adjusted regression coefficient	Deterioration	Increasing	R
					Anxiety	Occupation	Adjusted regression coefficient	Job insecurity high	Increasing	R
						Income	Adjusted regression coefficient	Deterioration	Increasing	R
Duarte and Jimenez-Molina, 2022 (49)	CL	*n* = 766	Cohort	May 2020 – Oct 2020	Depression & anxiety	Income	Regression coefficient	Lowest	Decreasing	R
						Education	Regression coefficient	Highest	Persistent	R
Ebrahimi et al., 2022 (50)	NO	*n* = 4,361	Cohort	Mar 2020 – Aug 2021	Depression	Education	Prevalence rate	Lowest	Decreasing	R
Ettman et al., 2020 (51)	USA	*n* = 6,506	Repeated cross-sectional	2017 – Apr 2020	Depression	Education	Prevalence rate, odds ratio	Lowest	Persistent	A, R
						Income	Prevalence rate, odds ratio	Lowest	Increasing	A, R
Ettman et al., 2022 (52)	USA	*n* = 1,441 (T1); *n* = 1,161 (T2)	Cohort (panel)	Mar 2020 – Apr 2021	Depression	Education	Weighted prevalence rate, adjusted odds ratio	Second lowest	Persistent	A, R
						Income	Weighted prevalence rate, adjusted odds Ratio	Lowest	Increasing	A, R
Fancourt et al., 2021 (53)	GB	*n* = 36,520	Cohort	Mar 2020 – Aug 2020	Depression	Education	Weighted prevalence rate	Middle	Decreasing	A
						Income	Weighted prevalence rate	Lowest	Persistent	A
					Anxiety	Education	Weighted prevalence rate	Middle	Decreasing	A
						Income	Weighted prevalence rate	Lowest	Persistent	A
Gigantesco et al., 2022 (54)	IT	*n* = 55,974	Repeated cross-sectional	Jan 2018 – Mar 2020	Depression	Occupation	Prevalence rate ratio	Unemployed	Decreasing	R
						Education	Prevalence rate ratio	Lowest	Decreasing	R
						Income	Prevalence rate ratio	Economic difficulties some/many	Increasing	R
				Mar 2020 – Dec 2020		Occupation	Prevalence rate ratio	Unemployed and temporarily employed	Persistent	R
						Education	Prevalence rate ratio	Lowest	Increasing	R
						Income	Prevalence rate ratio	Economic difficulties some/many	Decreasing	R
Goodwin et al., 2022 (55)	USA	*n* = 311,069	Repeated cross-sectional	2015–2020	Depression	Education	Prevalence rate (unadjusted and adjusted)	Middle	Persistent	A
						Income	Prevalence rate (unadjusted and adjusted)	Lowest	Persistent	A
Hajek et al., 2022 (56)	DE, GB, DK, NL, FR, PT, IT, ES	*n* = 16,351	Cohort	Jun 2021 – Jan 2022	Depression	Occupation	Prevalence rates, linear fe regression coefficients	Food retail	Persistent	A, R
						Education	Prevalence rates, linear FE regression coefficients	Highest	Persistent	A, R
						Income	Prevalence rates, linear FE regression coefficients	With great difficulty	Increasing	A, R
					Anxiety	Occupation	Prevalence rates, linear fe regression coefficients	Health-related sector in and research	Persistent	A, R
						Education	Prevalence rates, linear FE regression coefficients	Highest	Persistent	A, R
						Income	Prevalence rates, linear FE regression coefficients	With great difficulty	Increasing	A, R
Hajek et al., 2022 (57)	DE, GB, DK, NL, FR, PT, IT	*n* = 14,225	Cohort	Nov 2020 – Apr 2021	Depression	Education	Prevalence rates, odds ratios	Highest	Persistent	A, R
						Income	Prevalence rates, odds ratios	With great difficulty	Increasing	A, R
					Anxiety	Education	Prevalence rates, odds ratios	Highest	Persistent	A, R
						Income	Prevalence rates, odds ratios	With great difficulty	Increasing	A, R
Hertz-Palmor et al., 2021 (58)	USA, IL	*n* = 4,171	Cohort	Mar 2020 – Jun 2020	Depression	Income	Predicted increase in symptom load (models are adjusted for age, sex, relationship status, income, and country of origin)	Highest income loss	Increasing	R
Hou et al., 2021 (59)	HK	*n* = 6,029	Repeated cross-sectional	Feb 2020 – May 2020	Depression	Education	Adjusted odds ratio	From middle to lowest	Increasing	R
						Income	Adjusted odds ratio	From middle/highest to middle	Persistent	R
						Occupation	Adjusted odds ratio	Unemployed	Increasing	R
Jeong et al., 2022 (60)	KR	*n* = 30,359	Repeated cross-sectional	2016–2020	Depression	Occupation	Weighted prevalence rates	Unemployed	Increasing	A
						Income	Weighted prevalence rates	Lowest	Decreasing	A
Kessler et al., 2022 (61)	USA	*n* = 8,724	Repeated cross-sectional	Mar 2017 – Dec 2020	Depression & anxiety	Occupation	Prevalence rate, adjusted risk difference	Unemployed (long-term)	Decreasing	A
Khaled et al., 2022 (62)	QA	*n* = 6,064	Repeated cross-sectional	2017 – Jan 2021	Depression	Occupation	Average marginal effects and differences	Employed	Increasing	R
						Education	Average marginal effects and differences	Lowest	Persistent	R
					Anxiety	Occupation	Average marginal effects and differences	Unemployed	Persistent	R
						Education	Average marginal effects and differences	Lowest	Persistent	R
Kimhi et al., 2021 (10)	IL	*n* = 804	Cohort	May 2020 – Oct 2020	Anxiety	Income	Correlations, conditional (adjusted) trajectories	Lowest	Persistent	R
						Education	Correlations, latent growth mixture modeling	Middle	Persistent	R
						Occupation	Correlations, conditional (adjusted) trajectories	Economic difficulties	Increasing	R
					Depression	Income	Correlations, conditional (adjusted) trajectories	Lowest	Persistent	R
						Education	Correlations, conditional (adjusted) trajectories	Lowest	Persistent	R
						Occupation	Correlations, conditional (adjusted) trajectories	Economic difficulties	Increasing	R
König et al., 2023 (63)	DE,GB, DK,NL, FR,PT, IT	*n* = 7,160 (wave 1) – *n* = 7,300 (wave 9)	Cohort	Apr 2020 – Jan 2022	Depression & anxiety	Education	Prevalence rates, odds ratio	Lowest	Increasing	R
						Occupation	Prevalence rates, odds ratio	Food retail	Persistent	R
						Income	Prevalence rates, odds ratio	With great difficulty	Increasing	R
Kwong et al., 2021 (64)	GB	*n* = 10,659	Cohort	2011 – May 2020	Depression	Education	Associations estimates adjusted for the most recent pre-pandemic assessment of depression, sex, age and when the covid-19 questionnaire was completed	Lowest	Increasing	R						
						Income	Associations estimates adjusted for the most recent pre-pandemic assessment of depression, sex, age and when the COVID-19 questionnaire was completed	Lowest	Increasing	R
						Income	Associations estimates adjusted for the most recent pre-pandemic assessment of depression, sex, age and when the COVID-19 questionnaire was completed	Highest financial problems	Increasing	R
						Deprivation	Associations estimates adjusted for the most recent pre-pandemic assessment of depression, sex, age and when the COVID-19 questionnaire was completed	Worst deprivation status	Increasing	R
				1999 – May 2020	Anxiety	Education	Associations estimates adjusted for the most recent pre-pandemic assessment of depression, sex, age and when the covid-19 questionnaire was completed	Lowest	Increasing	R
						Income	Associations estimates adjusted for the most recent pre-pandemic assessment of depression, sex, age and when the COVID-19 questionnaire was completed	Lowest	Increasing	R
						Income	Associations estimates adjusted for the most recent pre-pandemic assessment of depression, sex, age and when the COVID-19 questionnaire was completed	Highest financial problems	Increasing	R
						Deprivation	Associations estimates adjusted for the most recent pre-pandemic assessment of depression, sex, age and when the COVID-19 questionnaire was completed	Worst deprivation status	Increasing	R
Lai et al., 2022 (65)	HK	*n* = 3,146	Repeated cross-sectional	Jul 2019 – Jul 2020	Depression	Education	Adjusted odds ratios	Lowest	Crossover	R
						Income	Adjusted odds ratios	Lowest	Persistent	R
						Occupation	Adjusted odds ratios	Employed	Crossover	R
Lee and Singh, 2021 (9)	USA	*n* = 1,144,405	Repeated cross-sectional	Apr 2020 – May 2021	Depression	Education	Weighted prevalence rate, unadjusted and adjusted odds ratio	Lowest	Increasing	A
						Income	Weighted prevalence, unadjusted and adjusted odds ratio	Lowest	Increasing	A
Lim et al., 2022 (66)	AU, GB,USA	*n* = 1,562	Cohort	Mar 2020 – Jul 2020	Depression	Occupation	Predictors of change	N.A.	Persistent	A
						Income	Predictors of change	N.A.	Persistent	A
					Anxiety	Occupation	Predictors of change	N.A.	Increasing	A
						Income	Predictors of change	N.A.	Increasing	A
Lowe et al., 2023 (67)	CA	*n* = 280	Cohort	May 2020 – Jan 2021	Depression	Occupation	Association between pandemic-related aoccupational status change and anxiety	From not economically active to economically active	Crossover	R
					Anxiety	Occupation	Association between prepandemic occupational status and depressive symptoms	Loss of occupational status to no change	Crossover	R
Mangot-Sala et al., 2023 (68)	NL	*n* = 76,795	Cohort	Apr 2020 – Jul 2021	Depression & anxiety	Occupation	Predicted symptom prevalence	Occupationally disabled	Persistent	A
Mauz et al., 2023 (69)	DE	*n* = 45,102	Repeated cross-sectional	Mar 2019 – Sep 2022	Depression	Education	Estimated percentage of positive screens (standardised for age, sex)	Lowest	Persistent	A, R
				Mar 2021 – Jun 2022	Anxiety	Education	Estimated percentage of positive screens (standardised for age, sex)	Lowest	Persistent	A, R
Min et al., 2022 (70)	KR	*n* = 915,089	Repeated cross-sectional	2017–2020	Depression	Education	Prevalence rate, adjusted odds ratios	Lowest	Decreasing	A, R
						Occupation	Prevalence rate, adjusted odds ratios	Unemployed	Decreasing	A, R
						Income	Prevalence rate, adjusted odds ratios	Lowest	Decreasing	A, R
Moreno-Agostino et al., 2022 (71)	GB	*n* = 26,772	Cohort	May 2020 – Mar 2021	Anxiety	Income	Unadjusted and adjusted marginal mean estimates	Lowest and second lowest (depending on cohort)	Persistent	A
					Depression	Income	Unadjusted and adjusted marginal mean estimates	Lowest and highest (depending on cohort)	Persistent	A
Parsons et al., 2022 (72)	GB	*n* = 34,465	Cohort	Apr 2020 – Apr 2021	Depression	Occupation	Predictors of class membership/ symptom trajectories	Unemployed	Increasing	R
					Anxiety	Occupation	Predictors of class membership/ symptom trajectories	Unemployed	Persistent	R
Petersen et al., 2022 (73)	DE	*n* = 10,250	Cohort	Okt 2020 – Mar 2021	Depression	Income	Prevalence differences	Lowest	Decreasing	R
					Anxiety	Income	Prevalence differences	Lowest	Persistent	R
Perez et al., 2021 (74)	PT	*n* = 748	Cohort	Mar 2020 – May 2020	Depression	Occupation	Linear regression models coefficients	Unemployed	Persistent	R
					Anxiety	Occupation	Linear regression models coefficients	Unemployed	Increasing	R
Probst-Hensch et al., 2023 (75)	SZ	*n* = 6,396	Cohort	Jul 2020 – Apr 2021	Depression	Income	Clusters of trajectories, group-based trajectory models (gbtm)	Lowest	Increasing	R
						Education	Clusters of trajectories, group-based trajectory Models (GBTM)	Lowest	Persistent	R
Qi et al., 2022 (76)	NL	*n* = 167,729	Cohort	2014 – Aug 2020	Depression	Income	Adjusted odds ratios for trajectories	Lowest	Increasing	R
						Education	Adjusted odds ratios for trajectories	Lowest	Increasing	R
						Occupation	Adjusted odds ratios for trajectories	Highest	Decreasing	R
					Anxiety	Income	Adjusted odds ratios for trajectories	Lowest	Increasing	R
						Education	Adjusted odds ratios for trajectories	Lowest	Decreasing	R
						Occupation	Adjusted odds ratios for trajectories	Highest	Decreasing	R
Ribeiro et al., 2021 (77)	LU	*n* = 1,756	Cohort	Apr 2020 – May 2020	Depression	Education	Linear regression coefficients (controlling for age, sex, education, previous psychological diagnostic)	Lowest	Increasing	R
						Occupation	Linear regression coefficients (controlling for age, sex, education, previous psychological diagnostic)	Permanently sick or disabled	Decreasing	R
						Income	Linear regression coefficients (controlling for age, sex, education, previous psychological diagnostic)	Lowest	Persistent	R					
					Anxiety	Education	Linear regression coefficients (controlling for age, sex, education, previous psychological diagnostic)	Lowest	Increasing	R
						Occupation	Linear regression coefficients (controlling for age, sex, education, previous psychological diagnostic)	Permanently sick or disabled	Persistent	R
						Income	Linear regression coefficients (controlling for age, sex, education, previous psychological diagnostic)	Lowest	Increasing	R
Riehm et al., 2021 (78)	USA	*n* = 6,901	Cohort	Mar 2020 – Aug 2020	Depression & anxiety	Income	Predicted probabilities, odds ratio (stratified by socioeconomic characteristics)	Lowest	Persistent	R
Robinson and Daly, 2021 (79)	USA	*n* = 7,138	Cohort	Mar 2020 – Jun 2020	Depression & anxiety	Income	Regression estimates of increase/decrease [adjusted for covariates (participant age, sex, race/ethnicity)]	Lowest	Increasing	R
						Occupation	Regression estimates of increase/decrease [adjusted for covariates (participant age, sex, race/ethnicity, household income)]	Unemployed	Increasing	R
Saunders et al., 2022 (30)	GB	*n* = 21,938	Cohort	Mar 2020 – Jul 2020	Anxiety	Education	Relative risk ratio for trajectory classes	Lowest	Increasing	R
						Income	Relative risk ratio for trajectory classes	Lowest	Increasing	R
					Depression	Education	Relative risk ratio for trajectory classes	Middle	Increasing	R
						Income	Relative risk ratio for trajectory classes	Lowest	Increasing	R
Shuster et al., 2021 (80)	USA	*n* = 1,512	Cohort	Apr 2020 – Jun 2020	Anxiety	Income	Mixed effects linear regression coefficients	Lowest	Increasing	A
					Depression	Income	Mixed effects linear regression coefficients	Lowest	Increasing	A
Tao et al., 2023 (81)	HK	*n* = 1,333	Cohort	Feb 2020 – Feb 2022	Depression	Education	Odds ratios of conditional growth mixture modeling trjaectories	Highest	Persistent	R
						Occupation	Odds ratios of conditional growth mixture modeling trjaectories	Dependent individuals	Persistent	R
						Income	Odds ratios of conditional growth mixture modeling trjaectories	Highest	Persistent	R
					Anxiety	Education	Odds ratios of conditional growth mixture modeling trjaectories	Lowest	Persistent	R
						Occupation	Odds ratios of conditional growth mixture modeling trjaectories	Employed	Persistent	R
						Income	Odds ratios of conditional growth mixture modeling trjaectories	Second lowest	Persistent	R
Twenge et al., 2021 (82)	USA	*n* = 1,291,943	Repeated cross-sectional	2019 – Apr 2020	Depression	Education	Prevalence rate, relative risk	Lowest	Increasing	A, R
						Income	Prevalence rate, relative risk	Lowest and second lowest	Persistent	A, R
						Occupation	Prevalence rate, relative risk	Caregiver/ disabled/ student	Increasing	A, R
					Anxiety	Education	Prevalence rate, relative risk	Lowest and second lowest	Increasing	A, R
						Income	Prevalence rate, relative risk	Lowest	Increasing	A, R
						Occupation	Prevalence rate, relative risk	Caregiver/ disabled/ student	Increasing	A, R
				Apr 2020 – Sep 2020	Depression	Education	Prevalence rate, relative risk	Lowest and second lowest	Decreasing	A, R
						Income	Prevalence rate, relative risk	Lowest	Decreasing	A, R
						Occupation	Prevalence rate, relative risk	Unemployed	Increasing	A, R
					Anxiety	Education	Prevalence rate, relative risk	Lowest and second lowest	Decreasing	A, R
						Income	Prevalence rate, relative risk	Lowest	Decreasing	A, R
						Occupation	Prevalence rate, relative risk	Unemployed	Increasing	A, R
Vahratian et al., 2021 (29)	USA	*n* = 790,633	Repeated cross-sectional	Aug 2020 – Feb 2021	Depression & anxiety	Education	Weighted percentage (adjusted for age, sex, race/ethnicity)	Lowest	Increasing	A
van der Velden et al., 2020 (83)	NL	*n* = 2,983	Cohort (panel)	Mar 2019 – Mar 2020	Depression & anxiety	Education	Prevalence rate, adjusted odds ratio (adjusted for all other predictors, e.g., age, gender, ethnicity)	Lowest	Persistent	A, R
						Occupation	Prevalence rate, adjusted odds ratio (adjusted for all other predictors, e.g., age, gender, ethnicity)	(partial) work dis.	Increasing	A, R
van der Velden et al., 2022 (84)	NL	*n* = 4,064	Cohort (panel)	Nov 2019 – Dec 2020	Depression & anxiety	Education	Prevalence rate, adjusted odds ratio (adjusted for all other predictors, e.g., age, gender, ethnicity)	Lowest	Persistent	A, R
						Occupation	Prevalence rate, adjusted odds ratio (adjusted for all other predictors, e.g., age, gender, ethnicity)	Unemployed	Persistent	A, R
Vancea and Apostol, 2021 (28)	RO	*n* = 1,126	Repeated cross-sectional	May 2020 – Nov 2020	Anxiety	Education	Odds ratio of binary logistic regression	Lowest	Increasing	A, R
					Depression	Education	Odds ratio of binary logistic regression	Lowest	Decreasing	A, R
Wanberg et al., 2022 (11)	USA	*n* = 1,143	Cohort (Panel)	Apr 2019 – Apr 2020	Depression	Education	Estimates of unstandardized structural path coefficients predicting change in depressive symptoms	Lowest	Decreasing	R
						Income	Estimates of unstandardized structural path coefficients predicting change in depressive symptoms	Lowest	Persistent	R
Yamamoto et al., 2022 (85)	JP	*n* = 6,112	Cohort	May 2020 – May 2021	Depression	Income	Prevalence rates	Lowest	Decreasing	A
Zhao et al., 2020 (86)	HK	*n* = 9,588	Repeated cross-sectional	2016 – Apr 2020	Depression	Education	Adjusted odds ratios (adjusting for sex, age, and marital status)	N.A.	Persistent	R
					Anxiety	Education	Adjusted odds ratios (adjusting for sex, age, and marital status)	N.A.	Increasing	R
Zhou et al., 2020 (87)	USA	*n* = 442	Cohort	Apr 2020 – May 2020	Depression	Education	Simple/multiple regression coefficients, standardised	N.A.	Persistent	R
						Occupation	Simple/multiple regression coefficients, standardised	N.A.	Persistent	R
					Anxiety	Education	Simple/multiple regression coefficients, standardised	N.A.	Persistent	R
						Occupation	Simple/multiple regression coefficients, standardised	N.A.	Persistent	R

Countries: Australia (AU), Canada (CA), Chile (CL), Denmark (DK), France (FR), Germany (DE), Great Britain (GB), Hong Kong (HK), Italy (IT), Israel (IL), Japan (JP), Luxembourg (LU), Norway (NO), Netherlands (NL), Portugal (PT), Qatar (QA), Romania (RO), South Korea (KR), Spain (ES), Switzerland (SZ), United States of America (USA); Measurement: Relative (R), Absolute (A).

Most of the included studies came from the USA (*n* = 14). 5 studies were conducted in the UK and 4 were from the Netherlands and Hong Kong. Three studies were conducted in Germany and Korea, respectively. One study was conducted in each of the following countries: Norway, Italy, Romania, Luxembourg, Switzerland, Portugal, Canada, Chile, Qatar, Israel and Japan. In addition, there were 2 cross-continental studies and 3 studies relating to multiple European countries.

Of the included studies, 32 were cohort studies and 17 were repeated cross-sectional studies. Six studies were published in 2020, 14 in 2021, 23 in 2022, and 6 in 2023.

The size of the sample populations varied substantially with a mean number of participants of 143,222 and a median number of participants of 7,160.

### Socioeconomic data

A comprehensive overview of the socioeconomic indicators used in the included studies can be found in Supplementary material S3. Income (*n* = 54) and education (*n* = 52) were the most frequently used socioeconomic indicators. Occupation, or employment, was used in 41 analyses. Two analyses assessed the SES by using the Index of Multiple Deprivation (IMD). Socioeconomic inequalities were most frequently assessed via relative measures (*n* = 90, 60.4%). In 22 (7.4%) analyses, only absolute measures and in 37 (24.8%) analyses, both relative and absolute measures were presented.

### Outcome measures

The scores used to measure symptoms of depression and anxiety disorders are shown in Supplementary material S4. Among the included studies, depression was the most frequently investigated outcome (*n* = 38). 24 studies investigated anxiety as an outcome. No study exclusively examined anxiety. In 24 studies, both depressive symptoms and symptoms of anxiety were assessed independently, and 11 studies measured a combined outcome of depression and anxiety.

The instrument most frequently used to measure symptoms of depression was the “Patient Health Questionnaire (PHQ)-9” (*n* = 16), and the second most used was the PHQ-2 (*n* = 8), a short version of the PHQ-9 (88). The “Depression Anxiety Stress Scale (DASS)-21 depression subscale” (*n* = 3) was used third most frequently.

To measure anxiety, the “Generalised Anxiety Disorder Scale (GAD)-2” (*n* = 8) was commonly used as an abbreviation of GAD-7 (88). The GAD-7 itself was used in seven studies. The DASS-21 anxiety subscale (*n* = 2) was used third most frequently.

If symptoms of depression and anxiety were measured in combination, the most frequently used instrument was the PHQ-4 (*n* = 6). All other instruments were used only once.

For the PHQ-9 and GAD-7 instruments, cut-off scores greater than or equal to 10 were used, or the cut-off score was not reported. For the short forms of these instruments, the PHQ-2 and GAD-2 questionnaires, cut-off scores ≥3 were used or not reported. The cut-off score for the PHQ-4 was generally ≥6, except for two studies, which set the cut-off score to 3 (78) or compared the average score over time (47).

### Observation periods

Most studies (*n* = 31) used at least two comparison time points during the COVID-19 pandemic, while 15 studies compared at least one time point before the pandemic with at least one time point during the pandemic. In three studies, multiple time points were used for comparison, so that analyses were possible both before-to-during and during the COVID-19 pandemic (54, 69, 82). The earliest start of the observation period compared from before to during the pandemic in the included studies was in 1999, while the latest start was in November 2019. The earliest onset for a comparison period within the pandemic was March 2020, while the latest beginning was June 2021. All studies’ mean duration of the observation period was 736 days, with a median of 365 days and a range from 32 days to 20 years. Frequently, studies used data showing temporal dynamics based on monthly or yearly outcome rates. Multiple studies, however, compared several pandemic waves or specific time points associated with contextual factors, such as the introduction of containment orders. Additional details on the observation periods can be found in Supplementary material S5.

### Temporal dynamics of socioeconomic inequalities in symptoms of depression and anxiety

In almost all analyses, socially disadvantaged groups were most affected at baseline. Most often, the lowest category was the most affected, rarely the second-lowest category, and occasionally also the middle category of the respective component of SES. However, there were some exceptions. Three studies from Hong Kong (59, 65, 81), two cohort studies from Chile and the UK (49, 71) and two studies across several European countries (56, 57) showed heterogenous results regarding the most affected socioeconomic group at baseline.

**Figure 2:**
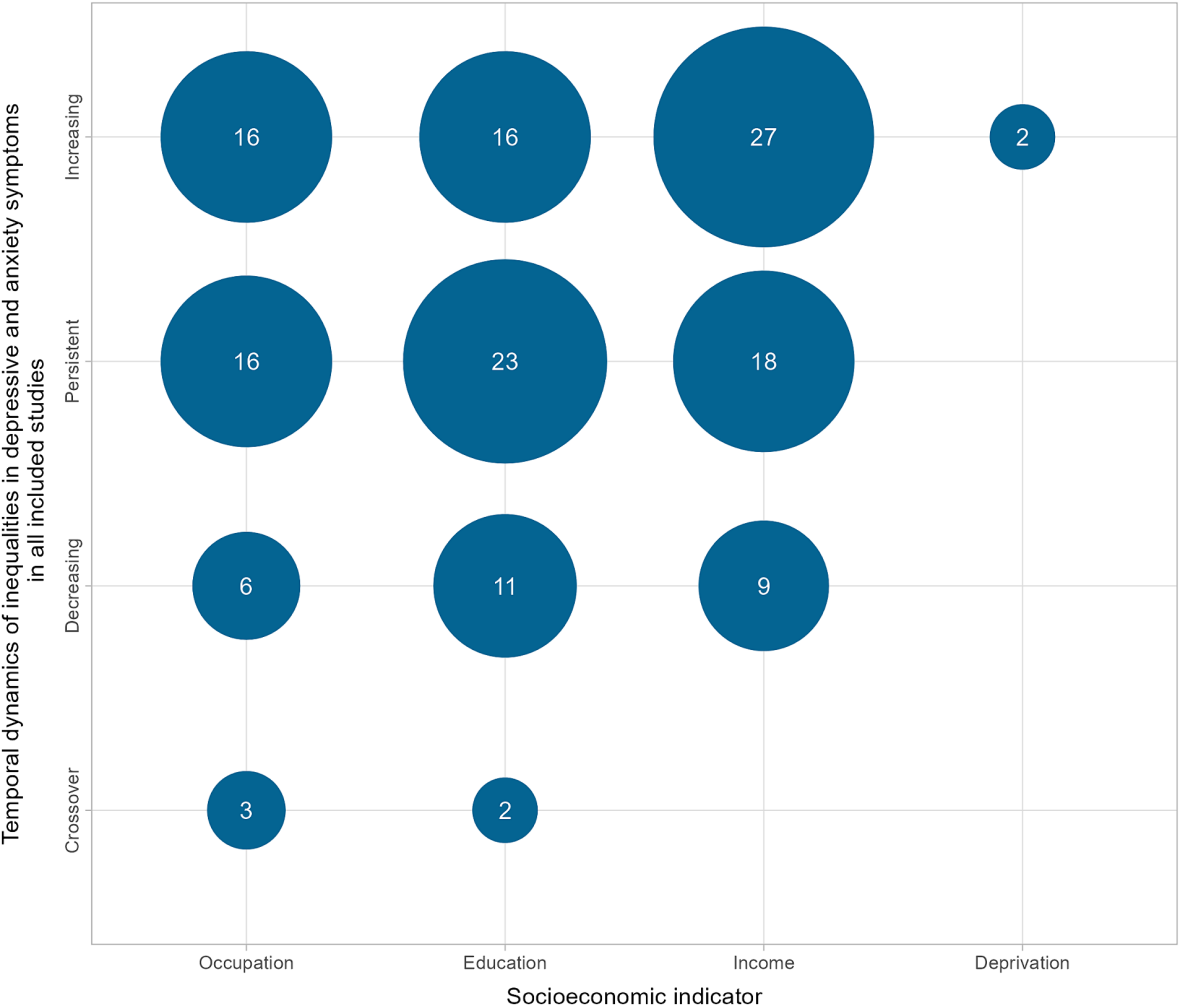
Number of analyses showing temporal dynamics of socioeconomic inequalities in depressive and anxiety symptoms in all included studies.

Of all analyses, 40.9% (*n* = 61) indicated increasing and 38.2% (*n* = 57) showed persistent inequalities. 17.4% (*n* = 26) found decreasing inequalities over time (Figure 2). Among the five analyses showing crossover dynamics in inequalities, four demonstrated a transition from initially higher prevalences in more deprived populations to higher prevalences in more affluent populations over time. One analysis showed crossover dynamics to the detriment of more deprived parts of the population. Considering the initial situation, most of the analyses (63.8%, *n* = 95) showed either persistent or increasing inequalities, with socioeconomically disadvantaged populations being the most affected.

### Temporal dynamics of inequalities before to during the COVID-19 pandemic

**Figure 3:**
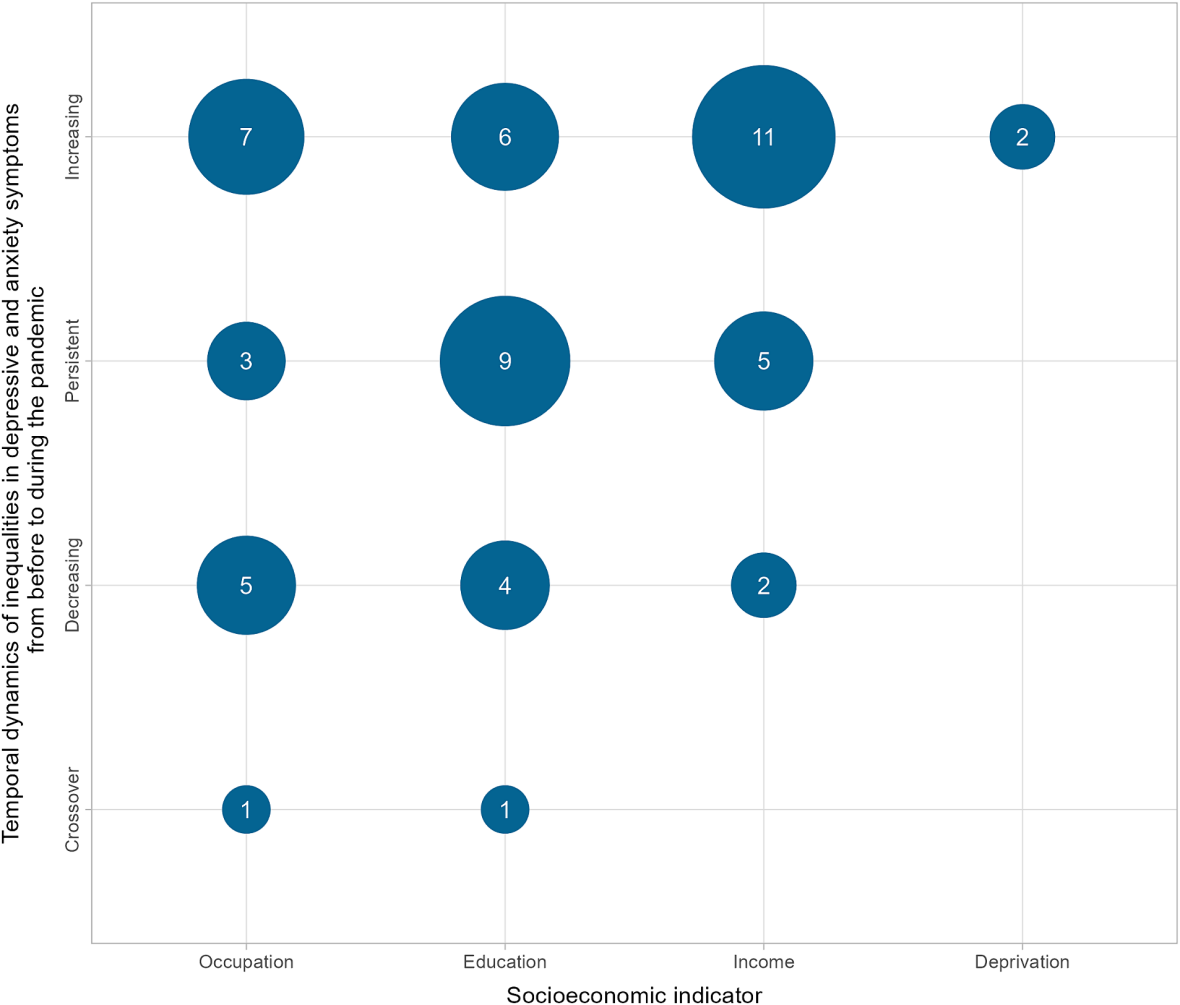
Number of analyses showing temporal dynamics in depressive and anxiety symptoms from before the pandemic to during the pandemic.

Similar to the overall proportions of all analyses, among the analyses that specifically focused on comparisons between periods before and during the COVID-19 pandemic (*n* = 56), 46.4% (*n* = 26) revealed increasing inequalities, 30.4% (*n* = 17) indicated persistent inequalities, 19.6% (*n* = 11) showed decreasing inequalities, and 3.6% (*n* = 2) found crossover dynamics over time (Figure 3).

Increasing and persistent inequalities combined accounted for more than three-quarters of the findings (76.8%). Specifically, with regard to income, the majority of the analyses (*n* = 11) indicated an increase in inequalities. In addition, we identified a trend of increasing or persistent inequalities during the early stages of the pandemic (March or April 2020) that negatively affected low SES groups (51, 82–86) in our more detailed analyses studies.

Upon closer examination of single countries, the USA stands out due to the highest number of included studies and contradictory results. Using the PHQ-9, Ettman et al. (51) showed that depressive symptoms tripled from 2017 to April 2020 in the USA, and low income and little savings were a risk for depressive symptoms in the initial stage of the COVID-19 pandemic. The population-representative survey study showed a stronger increase in depressive symptoms in the lowest and second-lowest income categories (51). In contrast, Wanberg et al. (11) came to different results, reporting that people with higher education had a higher risk of developing depressive symptoms in the USA between April 2019 and April 2020. The two studies differed in their pre-pandemic comparison date, screening tools, and methodology.

Regarding symptoms of common mental disorders and the dimension of educational attainment, a study conducted by Zhao et al. (86) in Hong Kong showed results similar to those of Ettman et al. in the USA. Specifically, Zhao et al. (86) analysed data on depression and anxiety symptoms from 2016 to April 2020 and found that individuals with low levels of formal education were particularly vulnerable to developing symptoms in the early stages of the pandemic. Mauz et al. (69) examined temporal trends in depressive symptoms based on educational attainment in Germany. Over the entire observation period from spring/summer 2019 to 2022, they observed an increase in the standardised proportions of individuals with a positive screen (69). A social gradient to the detriment of those with the lowest educational attainment was apparent throughout the whole observation period (69). In the high-level education group, there was a 5.2% increase (from 5.5%), while in the middle group, there was a 4.6% increase (from 10.6%), and in the low-level education group a 4.8% increase (from 16.2%) (69). Although all education groups had higher values at the end, the progression during the pandemic differed (69). The middle education group benefited at the beginning, and the higher education group was more affected as the pandemic progressed (69).

In the dimension of income or financial situation and inequality in symptoms of common mental-health disorders, a cohort study combining two different population cohorts from the UK ending in May 2020 found that socioeconomically disadvantaged people had higher levels of anxiety and depression, even when pre-pandemic levels were taken into account (64). Lower income, financial problems and deprivation were associated with a greater risk of symptoms of depression and anxiety. Anxiety symptoms exhibited a more profound increase compared to depressive symptoms. This surge was attributed to the uncertainty and sudden disruptions in daily life, coupled with health-related apprehensions (64).

### Temporal dynamics of inequalities during the COVID-19 pandemic

**Figure 4:**
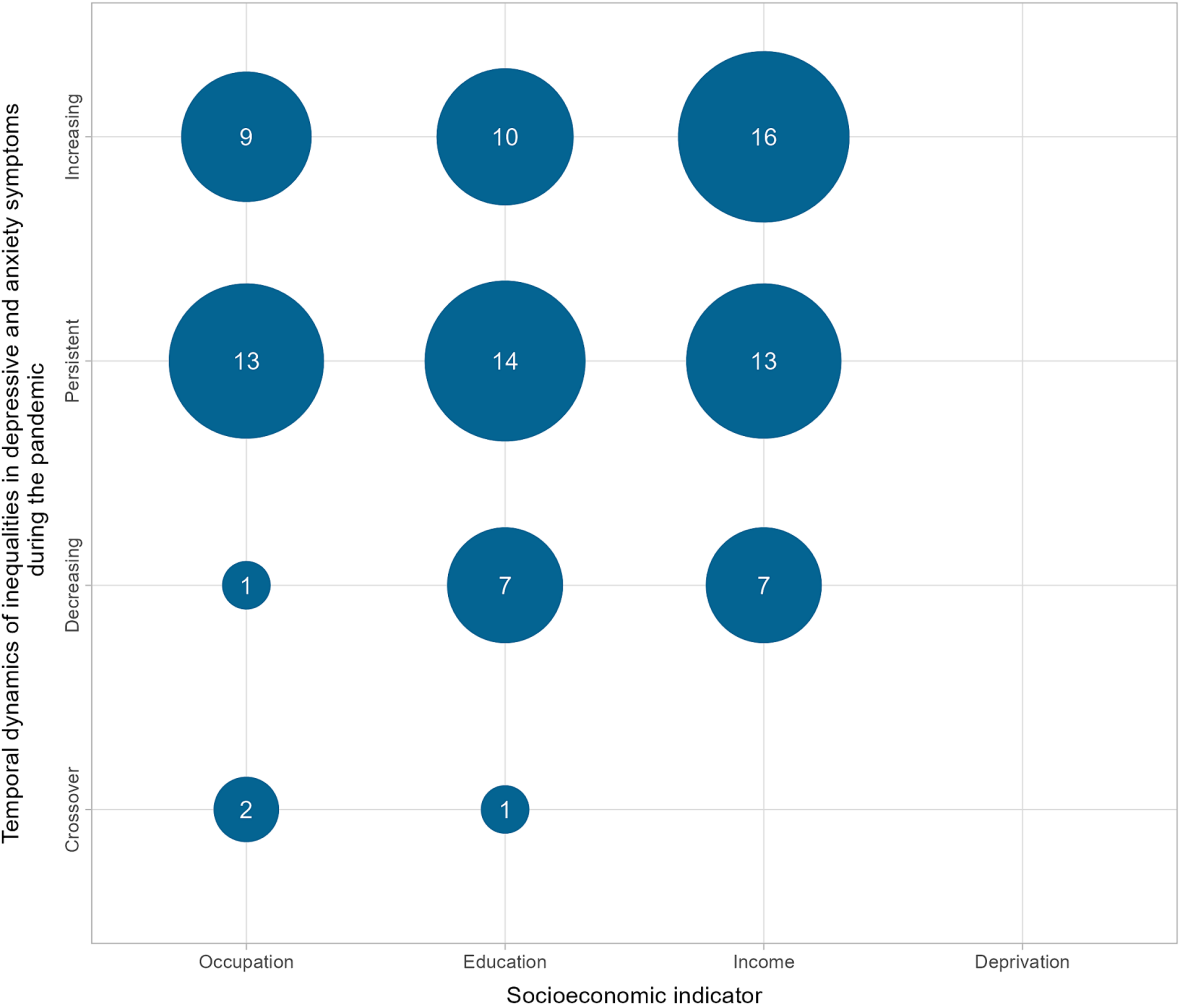
Number of analyses showing temporal dynamics of socioeconomic inequalities in depressive and anxiety symptoms during the pandemic.

Among the analyses investigating the dynamics of socioeconomic inequalities in symptoms of common mental disorders during the COVID-19 pandemic (*n* = 93), 43.0% (*n* = 40) showed persistent inequalities, 37.6% (*n* = 35) increasing inequalities, 16.1% (*n* = 15) decreasing inequalities, and 3.2% (*n* = 3) crossover dynamics (Figure 4).

Regarding symptoms of common mental disorders and the dimension of educational attainment, there were contrasting results in the USA, highlighted in the following section. Educational inequalities in depressive symptoms increased in the USA in May 2020 and then stabilised, according to Coley and Baum (46). In the case of symptoms of anxiety disorders, on the other hand, after an increase in May 2020, there was a decrease in educational inequality in autumn 2020 (46). Twenge et al. (82) reported that the relative increase in anxiety and depression in the USA was greater among those with higher education and income. However, those with lower education and income still had a higher prevalence (82). By contrast, Vahratian et al. (29) reported that the percentage of adults who had symptoms of anxiety disorder and depression increased during the pandemic, as did unmet mental-health needs. The most significant increases were among those aged between 18 and 29 and those with less than a high-school education (29).

In the dimension of income or financial situation and inequality in symptoms of common mental-health disorders, most studies concluded that inequalities have worsened or remained unchanged. In the UK, Saunders et al. (30) reported that from March to July 2020, individuals with lower incomes had higher symptoms at baseline, which then increased steadily during and after the relaxation of restrictions. Between May 2020 and February/March 2021, Moreno-Agostino et al. (71) examined self-reported financial difficulties, depression and anxiety in five existing UK adult cohorts. Large inequalities were observed and remained constant throughout the examined period (71). This finding aligned with previous research that found large inequalities employing various indicators of household economic conditions (16, 30, 53, 64). Riehm et al. (78) conducted a longitudinal study examining mental distress (PHQ-4) in a nationally representative sample of USA adults from March to August 2020. Prevalence of mental distress reached its highest point in mid-April to early May 2020 and subsequently declined (78). The trajectories of mental distress over the duration of the 4.5 months study period displayed a general resemblance across various sociodemographic subgroups, one of the investigated socioeconomic indicators being the distinction between individuals living below or above the federal poverty line (78).

Fancourt et al. (53) demonstrated with a prospective longitudinal observational study that inequalities in the UK tended to converge over 20 weeks after the lockdown was announced but persisted until the end of the study period. 36,520 participants had at least three measurements at different time points, and their data were analysed using latent growth models. Numerous risk factors associated with worse mental-health outcomes, such as female gender, younger age and lower educational attainment, were prominently observable during the early stages of the lockdown (53). As mental health gradually improved, the inequalities between these vulnerable demographic groups and those without these risk factors diminished. However, the inequalities in mental health persisted, and vulnerable groups have remained at risk (53).

## Discussion

This is the first systematic scoping review of the international evidence on the temporal dynamics of socioeconomic inequalities in symptoms of depression and anxiety disorders during the COVID-19 pandemic. Trends became evident despite the marked heterogeneity of the studies in terms of observation periods, outcomes, socioeconomic indicators and methods. Overall, most analyses showed increasing or persistent inequality trends. In the narrative synthesis of the studies, we identified a trend of increasing inequalities during the early stage of the pandemic that negatively affected low SES groups (46, 50, 51, 64). During the pandemic, there were very heterogeneous developments, with persistent inequalities reported most frequently (46, 69, 71). Large inequalities were observed concerning income, including financial situation using different indicators of household economic situation (16, 30, 53, 64, 71, 75, 80).

The included studies discussed potential explanations for the observed trends. In the initial phase of the COVID-19 pandemic, socioeconomic inequality in symptoms of depression and anxiety increased in many analyses. One reason proposed was that segments of the population with lower SES were disproportionately affected, experiencing job losses, reduced working hours, having to continue to work despite increased exposure, and particularly suffering from the deteriorating economic conditions (9, 10). A Canadian study reported that a third of their sample reported losing household income or occupational status (67). Those who moved down the social gradient and those who were always disadvantaged had a higher prevalence of symptoms of mental disorders (58, 80, 89). Dragano et al. (48) described that mental health was negatively influenced by pandemic-related job loss, reduction in working hours without short-time allowance, increase in working hours, switch to home-based work, increased job insecurity, and a worsening financial situation. The increase in mental-health problems was significantly lower after statistically controlling for work-related changes and financial strain (48). This indicates that the mean increase in symptom severity during the pandemic was primarily due to increased occupational and financial stress (48). Most pandemic-related job changes, except for increased home office work (76), may have particularly affected individuals with a low SES (26, 59, 73). Financial strain might have particularly afflicted individuals with lower incomes, which, in accordance with the vulnerability-stress model, might have led to deteriorating mental health (16, 90).

As the COVID-19 pandemic progressed, there were reports of increased depressive or anxiety symptoms among population groups with higher SES. One cited reason was the prevalence of remote work conditions (11). Min et al. (70) suggested that differential access to information may have contributed to the fact that people with higher SES were increasingly affected by the pandemic. Previous studies reported that people were exposed to anxiety-provoking information via social media, and more frequent exposure to such information may have led to higher vulnerability to mental-health deterioration when the future was unpredictable (91). In this regard, young, middle-aged, highly educated and high-income individuals were more likely to be exposed to negative information from the internet and social media than the general population (70). In addition, in several studies, the COR theory (18) was invoked to rationalise the fact that individuals with higher SES experienced relatively greater losses (11, 81). Higher SES could have been associated with a greater loss of interpersonal resources, as individuals with higher SES were likelier to work at home during the pandemic than those with lower SES (11).

Even in high-income countries, substantial heterogeneity existed in initial conditions, outcomes and mitigation measures during the COVID-19 pandemic. Some authors have suggested that this variability could account for divergent findings and conclusions, emphasising the importance of caution when extrapolating results across nations (13, 77, 83). Maffly-Kipp et al. (13) concluded that the severity of the COVID-19 pandemic influenced the relationship between socioeconomic factors and mental-health outcomes. In addition, van der Velden et al. (83) mentioned that differences in unemployment rates in March and April 2020 differed drastically between the USA and the Netherlands and might explain differences in results. A study from Luxembourg mentioned that Luxembourg implemented many policy measures to mitigate the spread of the disease and its economic consequences (77). Therefore, the authors emphasised that the mental-health impact should not be underestimated in other less affluent countries that were more seriously affected (77). A rapid review from 2021 in Germany showed that mental health was stable or advantageous for low SES groups during the first phase of the pandemic (92). The pronounced heterogeneity between countries could explain the discrepancy between the rapid review (92) and this scoping review.

Some studies identified demographic subgroups at higher risk for mental-health problems during the COVID-19 pandemic. Concerning generational inequality, young adults were frequently mentioned as disproportionally vulnerable during the COVID-19 pandemic (29, 30, 50, 54, 55, 62, 66, 70, 71, 75, 77, 78, 87, 91, 93). This could be related to SES, as young people often have yet to reach a higher level of education, are still studying or in training, and consequently have a lower income (94). They are pursuing their education instead of working in a full-time paid job (94). For this age group, the literature discusses the greater overall disruption of life during the pandemic (57) against the background of the particularly great importance of social contacts with peers when leaving home. If we also take a look at family health, which is still important for young adults, a scoping review of mediators of health inequalities in children and adolescents pointed to the role of parental mental health, parenting practices, and the parent–child relationship, which could also serve as targets for interventions (95). The majority of studies suggested future pandemic-preparedness measures, including health-care access and targeted interventions for disadvantaged populations (53, 64, 71, 74, 76). Identifying at-risk populations and collecting data on subgroups enables targeted support, as highlighted by multiple studies (30, 66, 69, 86).

Both relative and absolute measures of inequality were presented in only 24.8% of the analyses from the included studies. Research should precisely assess the magnitude and changes in socioeconomic health inequalities by consistently employing summary metrics for absolute and relative inequalities (96). This approach helps prevent biased interpretations of health-inequality trends resulting from the selective reporting of inequality measures (96). In future cohort or cross-sectional study publications, it would be desirable to present relative and absolute measures to view the development in its entirety. The measurement instruments of depressive symptoms and symptoms of anxiety disorders and their corresponding cut-off values were relatively uniform using the PHQ-9 and GAD-7 and their short forms. By contrast, the study designs and methodology were very heterogeneous. Standardised study designs and methodology would be desirable to answer social-epidemiological questions. Subsequent investigations are imperative to quantitatively elucidate the extent of the correlation between socioeconomic indicators and symptoms of common mental disorders over time. Addressing this inquiry will necessitate forthcoming reviews to concentrate on narrower sets of socioeconomic indicators (e.g., household income categories), outcomes (e.g., PHQ-9, GAD-7) and periods (e.g., specific waves during the pandemic). Continuous, unified monitoring and comprehension of developments might help to develop more targeted support.

### Strengths and limitations

This is the first systematically conducted scoping review investigating the temporal dynamics of socioeconomic inequalities related to symptoms of depression and anxiety disorders during the COVID-19 pandemic in high-income countries. The systematic literature search contributed to achieving a high degree of comprehensiveness and ensured the replicability of the results. However, it could not be entirely ruled out that additional relevant records were perhaps obtained via supplementary databases or manual searches. Systematic assessment of methodological limitations of the included studies was not possible due to the heterogeneity of the studies, which might have introduced bias into the narrative synthesis (97). Nevertheless, this approach of a scoping review appeared most fitting considering the review’s objectives of compiling a first and comprehensive overview. Potential selection and publication bias are concerns due to the review’s exclusive focus on peer-reviewed publications. Studies that found null results were possibly neglected and not published. This bias could have been mitigated by incorporating non-peer-reviewed literature. However, since a structured assessment of evidence quality could not be executed (32), the eligibility criteria were confined to peer-reviewed articles to enhance the quality of the findings encompassed in the study. For reasons of comparability, this review was limited to high-income countries (38, 39), which should be borne in mind when interpreting the results. In addition, this study included only articles published in English or German. This approach can limit the generalisability of the results.

## Conclusion

This scoping review can contribute to improving preparedness for future pandemics or crises. It became apparent that social inequality in mental health increased or at least persisted in most cases to the disadvantage of the deprived population. Considering the temporal dynamics of mental-health inequalities can help prevent harmful effects on the mental health of specific population groups during different pandemic stages. Job security, income security, educational attainment and community-based initiatives, including targeted prevention and intervention programmes that consider diverse socioeconomic backgrounds, could be important targets for interventions to address health inequalities. Socioeconomic inequalities must be monitored to adapt prevention and intervention measures to specific phases. Reducing socioeconomic inequalities is crucial to improving population health and achieving equity in the face of a significant burden of common mental-health disorders and existing inequalities.

## Data availability statement

The original contributions presented in the study are included in the article/Supplementary material, further inquiries can be directed to the corresponding author.

## Author contributions

KH: Conceptualization, Data curation, Formal analysis, Investigation, Methodology, Visualization, Writing – original draft, Writing – review & editing. FB: Conceptualization, Methodology, Visualization, Writing – review & editing. LW: Writing – review & editing, Data curation. EM: Writing – review & editing. CK: Writing – review & editing. JH: Writing – review & editing. BW: Conceptualization, Methodology, Supervision, Writing – review & editing.
