# Supplementary material for: Temporal dynamics of socioeconomic inequalities in depressive and anxiety symptoms during the COVID-19 pandemic: a scoping review
Source: Front Public Health. 2024 Jul 3;12:1397392. doi: 10.3389/fpubh.2024.1397392 (PMC11252079; doi:10.3389/fpubh.2024.1397392)
Supplement: Supplementary file 2 [file Data_Sheet_2.docx]

**Additional File 2**

Database-specific search strings

**Embase (OVID)**

**Concept of "socioeconomic inequalities"**

(socioeconomics OR socio-economics OR 'socioeconomic status' OR 'socio-economic status' OR 'socioeconomic position' OR 'socio-economic position' OR 'socioeconomic determinants' OR 'socio-economic determinants' OR 'socioeconomic class' OR 'socio-economic class' OR 'social class' OR 'social status' OR 'social position' OR 'social determinants' OR 'health disparities' OR 'health inequalities' OR 'social disparities' OR 'social inequalities' OR 'socioeconomic disparities' OR 'socioeconomic inequalities' OR 'socio-economic disparities' OR 'socio-economic inequalities' OR 'social gradient' OR 'socioeconomic gradient' OR 'socio-economic gradient' OR 'socioeconomic differences' OR 'socio-economic differences' OR 'social differences' OR 'health differences' OR ses OR sep OR income OR 'income inequality' OR education OR 'educational status' OR 'educational inequality' OR employment OR 'employment status' OR occupation OR occupational OR wealth* OR socioeconomics OR 'social status').ti,ab. OR exp socioeconomics/ OR exp 'social status'/

AND

**Concept "symptoms of common mental disorders"**

('common mental disorder*' OR 'common mental health disorder*' OR 'mental disorders' OR anxiety OR depression OR 'depressive symptoms' OR anxious OR depressive OR PTSD OR GAD OR phobia* OR psychopathology OR 'major depressive disorder' OR 'depressive episode' OR dysthymia OR depressed OR 'generalized anxiety disorder' OR 'panic disorder' OR 'social anxiety disorder' OR 'obsessive-compulsive disorder' OR 'depressed mood' OR 'post-traumatic stress disorder' OR 'mental-health' OR 'psychological health' OR 'mental disorder*' OR 'mentally disordered' OR 'mental health problem*' OR 'psychiatric disorder*' OR 'mental illness' OR 'mentally ill' OR 'mental sickness' OR 'mentally sick' OR 'mental disease' OR 'mental diseases' OR 'psychic health' OR 'psychiatric health' OR 'positive mental health' OR 'well-being' OR 'mental condition' OR 'psychological condition' OR 'mental constitution' OR 'mental health').ti,ab. OR exp mental disease/ OR exp 'anxiety disorder'/ OR exp anxiety/ OR exp depression/ OR exp 'mental health'/

AND

**Concept "SARS-CoV-2"**

('acute respiratory syndrome coronavirus 2' OR 'covid 19' OR ncov* OR covid* OR 'sars cov 2' OR 'sars-cov-2' OR 'sars coronavirus 2' OR 'severe acute respiratory syndrome cov 2' OR 'wuhan coronavirus' OR 'wuhan seafood market pneumonia virus' OR sars2 or '2019-ncov' OR 'hcov-19' OR 'novel 2019 coronavirus' OR '2019 novel coronavirus*' OR 'novel coronavirus 2019' OR '2019 novel human coronavirus*' OR 'human coronavirus 2019' OR 'coronavirus disease-19' OR 'corona virus disease-19' OR 'coronavirus disease 2019' OR 'corona virus disease 2019' OR '2019 coronavirus disease' OR 'novel coronavirus 2019*' OR 'novel coronavirus disease 2019' OR 'novel coronavirus infection 2019' OR '2019 corona virus disease' OR 'new coronavirus*' OR 'coronavirus outbreak' OR 'coronavirus epidemic' OR 'coronavirus pandemic' OR 'pandemic of coronavirus' OR 'coronavirus disease 2019' OR 'severe acute respiratory syndrome coronavirus 2').ti,ab. OR exp 'coronavirus disease 2019'/ OR exp 'severe acute respiratory syndrome coronavirus 2'/

AND

**Concept "longitudinal"**

(longitudinal* OR repeat* OR cohort OR trend* OR change OR dynamics OR 'time series' OR consecutive OR recurrent OR replicated OR sequential OR periodic OR prospective OR (before and during)).ti,ab. OR exp cohort analysis/

NOT

**Concept "Exclude Low- and middle-income countries"**

(afghanistan OR 'burkina faso' OR burundi OR 'central african republic' OR chad OR eritrea OR ethiopia OR gambia OR guinea OR 'guinea-bissau' OR 'north korea' OR liberia OR madagascar OR malawi OR mali OR mozambique OR niger OR rwanda OR 'sierra leone' OR somalia OR 'south sudan' OR sudan OR 'syrian arab republic' OR syria OR togo OR uganda OR yemen OR angola OR algeria OR bangladesh OR belize OR benin OR bhutan OR bolivia OR brazil OR 'cabo verde' OR cambodia OR cameroon OR comoros OR congo OR 'cote d`ivoire' OR djibouti OR egypt OR 'el salvador' OR eswatini OR ghana OR haiti OR honduras OR india OR indonesia OR iran OR kenya OR kiribati OR kyrgyzstan OR laos OR lesotho OR mauritania OR 'federated states of micronesia' OR mongolia OR morocco OR myanmar OR nepal OR nicaragua OR nigeria OR pakistan OR 'papua new guinea' OR philippines OR samoa OR 'sao tome' OR principe OR senegal OR 'solomon islands' OR 'south africa' OR 'sri lanka' OR tanzania OR tajikistan OR 'timor leste' OR tunisia OR ukraine OR uzbekistan OR vanuatu OR vietnam OR 'gaza strip palestine' OR zambia OR zimbabwe OR albania OR 'american samoa' OR argentina OR armenia OR azerbaijan OR belarus OR bosnia OR herzegovina OR botswana OR bulgaria OR colombia OR 'costa rica' OR cuba OR dominica OR 'dominican republic' OR 'equatorial guinea' OR ecuador OR fiji OR gabon OR grenada OR guatemala OR guyana OR iraq OR jamaica OR jordan OR kazakhstan OR kosovo OR lebanon OR libya OR malaysia OR maldives OR 'marshall islands' OR mauritius OR mexico OR moldova OR montenegro OR namibia OR 'north macedonia' OR palau OR paraguay OR peru OR russia OR 'russian federation' OR serbia OR 'saint lucia' OR 'saint vincent' OR 'the grenadines' OR suriname OR thailand OR tonga OR turkey OR turkmenistan OR tuvalu).ti,ab.

**Scopus**

**Concept of "socioeconomic inequalities"**

(TITLE-ABS ( socioeconomics OR socio-economics OR "socioeconomic status" OR "socio-economic status" OR "socioeconomic position" OR "socio-economic position" OR "socioeconomic determinants" OR "socio-economic determinants" OR "socioeconomic class" OR "socio-economic class" OR "social class" OR "social status" OR "social position" OR "social determinants" OR "health disparities" OR "health inequalities" OR "social disparities" OR "social inequalities" OR "socioeconomic disparities" OR "socioeconomic inequalities" OR "socio-economic disparities" OR "socio-economic inequalities" OR "social gradient" OR "socioeconomic gradient" OR "socio-economic gradient" OR "socioeconomic differences" OR "socio-economic differences" OR "social differences" OR "health differences" OR ses OR sep OR income OR "income inequality" OR education OR "educational status" OR "educational inequality" OR employment OR "employment status" OR occupation OR occupational OR wealth* OR "socioeconomics” OR “social status'") OR INDEXTERMS ( "social status" OR "socioeconomic factor" OR "social class" OR "socioeconomics" OR "Socioeconomic disparities in health"))

AND

**Concept of "symptoms of common mental disorders"**

(TITLE-ABS ( "common mental disorder*" OR "common mental health disorder*" OR "mental disorders" OR anxiety OR depression OR "depressive symptoms" OR anxious OR depressive OR "phobia*" OR "psychopathology" OR "major depressive disorder" OR "depressive episode" OR dysthymia OR depressed OR "generalized anxiety disorder" OR "panic disorder" OR "social anxiety disorder" OR "obsessive-compulsive disorder" OR "depressed mood" OR "post-traumatic stress disorder" OR "mental-health" OR "psychological health" OR "mental disorder*" OR "mentally disordered" OR "mental health problem*" OR "psychiatric disorder*" OR "mental illness" OR "mentally ill" OR "mental sickness" OR "mentally sick" OR "mental disease" OR "mental diseases" OR "psychic health" OR "psychiatric health" OR "positive mental health" OR "well-being" OR "mental condition" OR "psychological condition" OR "mental constitution" OR "mental health" ) OR INDEXTERMS (depression OR anxiety OR "depressive symptoms" OR "depressive disorder" OR "anxiety disorders"))

AND

**Concept of "SARS-CoV-2"**

(TITLE-ABS ( "acute respiratory syndrome coronavirus 2" OR "covid 19" OR ncov* OR covid* OR "sars cov 2" OR 'sars-cov-2' OR "sars coronavirus 2" OR "severe acute respiratory syndrome cov 2" OR "wuhan coronavirus" OR "wuhan seafood market pneumonia virus" OR sars2 OR '2019-ncov' OR 'hcov-19' OR "novel 2019 coronavirus" OR "2019 novel coronavirus*" OR "novel coronavirus 2019" OR "2019 novel human coronavirus*" OR "human coronavirus 2019" OR "coronavirus disease-19" OR "corona virus disease-19" OR "coronavirus disease 2019" OR "corona virus disease 2019" OR "2019 coronavirus disease" OR "novel coronavirus 2019*" OR "novel coronavirus disease 2019" OR "novel coronavirus infection 2019" OR "2019 corona virus disease" OR "new coronavirus*" OR "coronavirus outbreak" OR "coronavirus epidemic" OR "coronavirus pandemic" OR "pandemic of coronavirus" OR "coronavirus disease 2019" OR "severe acute respiratory syndrome coronavirus 2" ) OR INDEXTERMS ("COVID-19" OR "sars-cov-2"))

AND

**Concept "longitudinal"**

(TITLE-ABS (longitudinal* OR repeat* OR cohort OR trend* OR change OR dynamics OR "time series" OR "before and during" OR "consecutive" OR "recurrent" OR "replicated" OR "sequential" OR "periodic" OR "prospective") OR INDEXTERMS ("cohort analysis" OR "cohort analyses" OR "longitudinal studies"))

AND NOT

**Concept "Exclude Low- and middle-income countries"**

(TITLE-ABS ( afghanistan OR "burkina faso" OR burundi OR "central african republic" OR chad OR eritrea OR ethiopia OR gambia OR guinea OR 'guinea-bissau' OR "north korea" OR liberia OR madagascar OR malawi OR mali OR mozambique OR niger OR rwanda OR "sierra leone" OR somalia OR "south sudan" OR sudan OR "syrian arab republic" OR syria OR togo OR uganda OR yemen OR angola OR algeria OR bangladesh OR belize OR benin OR bhutan OR bolivia OR brazil OR "cabo verde" OR cambodia OR cameroon OR comoros OR congo OR "cote d`ivoire" OR djibouti OR egypt OR "el salvador" OR eswatini OR ghana OR haiti OR honduras OR india OR indonesia OR iran OR kenya OR kiribati OR kyrgyzstan OR laos OR lesotho OR mauritania OR "federated states of micronesia" OR mongolia OR morocco OR myanmar OR nepal OR nicaragua OR nigeria OR pakistan OR "papua new guinea" OR philippines OR samoa OR "sao tome" OR principe OR senegal OR "solomon islands" OR "south africa" OR "sri lanka" OR tanzania OR tajikistan OR "timor leste" OR tunisia OR ukraine OR uzbekistan OR vanuatu OR vietnam OR "gaza strip palestine" OR zambia OR zimbabwe OR albania OR "american samoa" OR argentina OR armenia OR azerbaijan OR belarus OR bosnia OR herzegovina OR botswana OR bulgaria OR colombia OR "costa rica" OR cuba OR dominica OR "dominican republic" OR "equatorial guinea" OR ecuador OR fiji OR gabon OR grenada OR guatemala OR guyana OR iraq OR jamaica OR jordan OR kazakhstan OR kosovo OR lebanon OR libya OR malaysia OR maldives OR "marshall islands" OR mauritius OR mexico OR moldova OR montenegro OR namibia OR "north macedonia" OR palau OR paraguay OR peru OR russia OR "russian federation" OR serbia OR "'saint lucia'" OR "saint vincent" OR "the grenadines" OR suriname OR thailand OR tonga OR turkey OR turkmenistan OR tuvalu ))

**PsycINFO (via EBSCO HOST)**

**Concept of "socioeconomic inequalities"**

(TI socioeconomics OR AB socioeconomics OR TI socio-economics OR AB socio-economics OR TI 'socioeconomic status' OR AB 'socioeconomic status' OR TI 'socio-economic status' OR AB 'socio-economic status' OR TI 'socioeconomic position' OR AB 'socioeconomic position' OR TI 'socio-economic position' OR AB 'socio-economic position' OR TI 'socioeconomic determinants' OR AB 'socioeconomic determinants' OR TI 'socio-economic determinants' OR AB 'socio-economic determinants' OR TI 'socioeconomic class' OR AB 'socioeconomic class' OR TI 'socio-economic class' OR AB 'socio-economic class' OR TI 'social class' OR AB 'social class' OR TI 'social status' OR AB 'social status' OR TI 'social position' OR AB 'social position' OR TI 'social determinants' OR AB 'social determinants' OR TI 'health disparities' OR AB 'health disparities' OR TI 'health inequalities' OR AB 'health inequalities' OR TI 'social disparities' OR AB 'social disparities' OR TI 'social inequalities' OR AB 'social inequalities' OR TI 'socioeconomic disparities' OR AB 'socioeconomic disparities' OR TI 'socioeconomic inequalities' OR AB 'socioeconomic inequalities' OR TI 'socio-economic disparities' OR AB 'socio-economic disparities' OR TI 'socio-economic inequalities' OR AB 'socio-economic inequalities' OR TI 'social gradient' OR AB 'social gradient' OR TI 'socioeconomic gradient' OR AB 'socioeconomic gradient' OR TI 'socio-economic gradient' OR AB 'socio-economic gradient' OR TI 'socioeconomic differences' OR AB 'socioeconomic differences' OR TI 'socio-economic differences' OR AB 'socio-economic differences' OR TI 'social differences' OR AB 'social differences' OR TI 'health differences' OR AB 'health differences' OR TI ses OR AB ses OR TI sep OR AB sep OR TI income OR AB income OR TI 'income inequality' OR AB 'income inequality' OR TI education OR AB education OR TI 'educational status' OR AB 'educational status' OR TI 'educational inequality' OR AB 'educational inequality' OR TI employment OR AB employment OR TI 'employment status' OR AB 'employment status' OR TI occupation OR AB occupation OR TI occupational OR AB occupational OR TI wealth* OR AB wealth* OR TI socioeconomics OR AB socioeconomics) OR (MA socioeconomic factors+) OR (MA social class+) OR (MA socioeconomic status+)

AND

**Concept of "symptoms of common mental disorder"**

(TI 'common mental disorder*' OR AB 'common mental disorder*' OR TI 'common mental health disorder*' OR AB 'common mental health disorder*' OR TI 'mental disorders' OR AB 'mental disorders' OR TI anxiety OR AB anxiety OR TI depression OR AB depression OR TI 'depressive symptoms' OR AB 'depressive symptoms' OR TI anxious OR AB anxious OR TI depressive OR AB depressive OR TI PTSD OR AB PTSD OR TI GAD OR AB GAD OR TI phobia* OR AB phobia* OR TI psychopathology OR AB psychopathology OR TI 'major depressive disorder' OR AB 'major depressive disorder' OR TI 'depressive episode' OR AB 'depressive episode' OR TI dysthymia OR AB dysthymia OR TI depressed OR AB depressed OR TI 'generalized anxiety disorder' OR AB 'generalized anxiety disorder' OR TI 'panic disorder' OR AB 'panic disorder' OR TI 'social anxiety disorder' OR AB 'social anxiety disorder' OR TI 'obsessive-compulsive disorder' OR AB 'obsessive-compulsive disorder' OR TI 'depressed mood' OR AB 'depressed mood' OR TI 'post-traumatic stress disorder' OR AB 'post-traumatic stress disorder' OR TI 'mental-health' OR AB 'mental-health' OR TI 'psychological health' OR AB 'psychological health' OR TI 'mental disorder*' OR AB 'mental disorder*' OR TI 'mentally disordered' OR AB 'mentally disordered' OR TI 'mental health problem*' OR AB 'mental health problem*' OR TI 'psychiatric disorder*' OR AB 'psychiatric disorder*' OR TI 'mental illness' OR AB 'mental illness' OR TI 'mentally ill' OR AB 'mentally ill' OR TI 'mental sickness' OR AB 'mental sickness' OR TI 'mentally sick' OR AB 'mentally sick' OR TI 'mental disease' OR AB 'mental disease' OR TI 'mental diseases' OR AB 'mental diseases' OR TI 'psychic health' OR AB 'psychic health' OR TI 'psychiatric health' OR AB 'psychiatric health' OR TI 'positive mental health' OR AB 'positive mental health' OR TI 'well-being' OR AB 'well-being' OR TI 'mental condition' OR AB 'mental condition' OR TI 'psychological condition' OR AB 'psychological condition' OR TI 'mental constitution' OR AB 'mental constitution' OR TI 'mental health' OR AB 'mental health') OR (MA anxiety screening+) OR (MA anxiety disorder+) OR (MA anxiety+) OR (MA depression Screening+) OR (MA major depression+)

AND

**Concept of "SARS-CoV-2"**

(TI 'acute respiratory syndrome coronavirus 2' OR AB 'acute respiratory syndrome coronavirus 2' OR TI 'covid 19' OR AB 'covid 19' OR TI ncov* OR AB ncov* OR TI covid* OR AB covid* OR TI 'sars cov 2' OR AB 'sars cov 2' OR TI 'sars-cov-2' OR AB 'sars-cov-2' OR TI 'sars coronavirus 2' OR AB 'sars coronavirus 2' OR TI 'severe acute respiratory syndrome cov 2' OR AB 'severe acute respiratory syndrome cov 2' OR TI 'wuhan coronavirus' OR AB 'wuhan coronavirus' OR TI 'wuhan seafood market pneumonia virus' OR AB 'wuhan seafood market pneumonia virus' OR TI sars2 OR AB sars2 OR TI '2019-ncov' OR AB '2019-ncov' OR TI 'hcov-19' OR AB 'hcov-19' OR TI 'novel 2019 coronavirus' OR AB 'novel 2019 coronavirus' OR TI '2019 novel coronavirus*' OR AB '2019 novel coronavirus*' OR TI 'novel coronavirus 2019' OR AB 'novel coronavirus 2019' OR TI '2019 novel human coronavirus*' OR AB '2019 novel human coronavirus*' OR TI 'human coronavirus 2019' OR AB 'human coronavirus 2019' OR TI 'coronavirus disease-19' OR AB 'coronavirus disease-19' OR TI 'corona virus disease-19' OR AB 'corona virus disease-19' OR TI 'coronavirus disease 2019' OR AB 'coronavirus disease 2019' OR TI 'corona virus disease 2019' OR AB 'corona virus disease 2019' OR TI '2019 coronavirus disease' OR AB '2019 coronavirus disease' OR TI 'novel coronavirus 2019*' OR AB 'novel coronavirus 2019*' OR TI 'novel coronavirus disease 2019' OR AB 'novel coronavirus disease 2019' OR TI 'novel coronavirus infection 2019' OR AB 'novel coronavirus infection 2019' OR TI '2019 corona virus disease' OR AB '2019 corona virus disease' OR TI 'new coronavirus*' OR AB 'new coronavirus*' OR TI 'coronavirus outbreak' OR AB 'coronavirus outbreak' OR TI 'coronavirus epidemic' OR AB 'coronavirus epidemic' OR TI 'coronavirus pandemic' OR AB 'coronavirus pandemic' OR TI 'pandemic of coronavirus' OR AB 'pandemic of coronavirus' OR TI 'coronavirus disease 2019' OR AB 'coronavirus disease 2019' OR TI 'severe acute respiratory syndrome coronavirus 2' OR AB 'severe acute respiratory syndrome coronavirus 2') OR (MA covid-19+)

AND

**Concept "longitudinal"**

(TI longitudinal* OR AB longitudinal* OR TI repeat* OR AB repeat* OR TI cohort OR AB cohort OR TI trend* OR AB trend* OR TI change OR AB change OR TI dynamics OR AB dynamics OR TI time series OR AB time series OR TI "Before and during" OR AB "Before and during" OR TI consecutive OR AB consecutive OR TI recurrent OR AB recurrent OR TI replicated OR AB replicated OR TI sequential OR AB sequential OR TI periodic OR AB periodic OR TI prospective OR AB prospective) OR (MA cohort analysis+)

NOT

**Concept "Exclude Low- and middle-income countries"**

TI (afghanistan OR 'burkina faso' OR burundi OR 'central african republic' OR chad OR eritrea OR ethiopia OR gambia OR guinea OR 'guinea-bissau' OR 'north korea' OR liberia OR madagascar OR malawi OR mali OR mozambique OR niger OR rwanda OR 'sierra leone' OR somalia OR 'south sudan' OR sudan OR 'syrian arab republic' OR syria OR togo OR uganda OR yemen OR angola OR algeria OR bangladesh OR belize OR benin OR bhutan OR bolivia OR brazil OR 'cabo verde' OR cambodia OR cameroon OR comoros OR congo OR 'cote d`ivoire' OR djibouti OR egypt OR 'el salvador' OR eswatini OR ghana OR haiti OR honduras OR india OR indonesia OR iran OR kenya OR kiribati OR kyrgyzstan OR laos OR lesotho OR mauritania OR 'federated states of micronesia' OR mongolia OR morocco OR myanmar OR nepal OR nicaragua OR nigeria OR pakistan OR 'papua new guinea' OR philippines OR samoa OR 'sao tome' OR principe OR senegal OR 'solomon islands' OR 'south africa' OR 'sri lanka' OR tanzania OR tajikistan OR 'timor leste' OR tunisia OR ukraine OR uzbekistan OR vanuatu OR vietnam OR 'gaza strip palestine' OR zambia OR zimbabwe OR albania OR 'american samoa' OR argentina OR armenia OR azerbaijan OR belarus OR bosnia OR herzegovina OR botswana OR bulgaria OR colombia OR 'costa rica' OR cuba OR dominica OR 'dominican republic' OR 'equatorial guinea' OR ecuador OR fiji OR gabon OR grenada OR guatemala OR guyana OR iraq OR jamaica OR jordan OR kazakhstan OR kosovo OR lebanon OR libya OR malaysia OR maldives OR 'marshall islands' OR mauritius OR mexico OR moldova OR montenegro OR namibia OR 'north macedonia' OR palau OR paraguay OR peru OR russia OR 'russian federation' OR serbia OR 'saint lucia' OR 'saint vincent' OR 'the grenadines' OR suriname OR thailand OR tonga OR turkey OR turkmenistan OR tuvalu) OR AB (afghanistan OR 'burkina faso' OR burundi OR 'central african republic' OR chad OR eritrea OR ethiopia OR gambia OR guinea OR 'guinea-bissau' OR 'north korea' OR liberia OR madagascar OR malawi OR mali OR mozambique OR niger OR rwanda OR 'sierra leone' OR somalia OR 'south sudan' OR sudan OR 'syrian arab republic' OR syria OR togo OR uganda OR yemen OR angola OR algeria OR bangladesh OR belize OR benin OR bhutan OR bolivia OR brazil OR 'cabo verde' OR cambodia OR cameroon OR comoros OR congo OR 'cote d`ivoire' OR djibouti OR egypt OR 'el salvador' OR eswatini OR ghana OR haiti OR honduras OR india OR indonesia OR iran OR kenya OR kiribati OR kyrgyzstan OR laos OR lesotho OR mauritania OR 'federated states of micronesia' OR mongolia OR morocco OR myanmar OR nepal OR nicaragua OR nigeria OR pakistan OR 'papua new guinea' OR philippines OR samoa OR 'sao tome' OR principe OR senegal OR 'solomon islands' OR 'south africa' OR 'sri lanka' OR tanzania OR tajikistan OR 'timor leste' OR tunisia OR ukraine OR uzbekistan OR vanuatu OR vietnam OR 'gaza strip palestine' OR zambia OR zimbabwe OR albania OR 'american samoa' OR argentina OR armenia OR azerbaijan OR belarus OR bosnia OR herzegovina OR botswana OR bulgaria OR colombia OR 'costa rica' OR cuba OR dominica OR 'dominican republic' OR 'equatorial guinea' OR ecuador OR fiji OR gabon OR grenada OR guatemala OR guyana OR iraq OR jamaica OR jordan OR kazakhstan OR kosovo OR lebanon OR libya OR malaysia OR maldives OR 'marshall islands' OR mauritius OR mexico OR moldova OR montenegro OR namibia OR 'north macedonia' OR palau OR paraguay OR peru OR russia OR 'russian federation' OR serbia OR 'saint lucia' OR 'saint vincent' OR 'the grenadines' OR suriname OR thailand OR tonga OR turkey OR turkmenistan OR tuvalu)
