# Supplementary material for: Temporal dynamics of socioeconomic inequalities in depressive and anxiety symptoms during the COVID-19 pandemic: a scoping review
Source: Front Public Health. 2024 Jul 3;12:1397392. doi: 10.3389/fpubh.2024.1397392 (PMC11252079; doi:10.3389/fpubh.2024.1397392)
Supplement: Supplementary file 3 [file Data_Sheet_3.docx]

**Additional file 3**

Socioeconomic indicators

**Table:** Socioeconomic indicators of the included analyses

| **Measures of SES** | **Measurement** | **Number of categories if applicable** | **Number of analyses** |
| --- | --- | --- | --- |
| **Income (n=54)** | Household income categories (n=15) | 2 | 3 |
|  |  | 3 | 4 |
|  |  | 4 | 3 |
|  |  | 5 | 3 |
|  |  | 6 | 1 |
|  |  | 17 | 1 |
|  | Household income quintiles (n=1) | 5 | 1 |
|  | Household income percentiles (n=2) | n.a. | 2 |
|  | Changes in financial situation (n=2) | 3 | 2 |
|  | Income loss (n=1) | 3-5 | 1 |
|  | Ln household income (n=1) | n.a. | 1 |
|  | Economic difficulties/ Financial problems/ perceived difficulties with income/ perceived financial risk (n=12) | 2 | 2 |
|  |  | 3 | 2 |
|  |  | 4 | 5 |
|  |  | Continuous variable | 3 |
|  | Income category (n=13) | 4 | 5 |
|  |  | 5 | 4 |
|  |  | 12 | 2 |
|  |  | Continuous variable | 2 |
|  | Family income category (n=2) | 5 | 2 |
|  | Financial status (n=2) | 2 | 2 |
|  | Risk of poverty/ Poverty line (n=3) | 2 | 1 |
|  |  | 6 | 2 |
| **Education (n=52)** | Educational categories (n=52) | 2 | 10 |
|  |  | 3 | 21 |
|  |  | 4 | 10 |
|  |  | 5 | 5 |
|  |  | 6 | 1 |
|  |  | 9 | 1 |
|  |  | n.a. | 2 |
|  |  | Continuous variable | 2 |
| **Occupation and Employment (n=41)** | Employment categories (n=31) | 2 | 9 |
|  |  | 3 | 8 |
|  |  | 4 | 7 |
|  |  | 5 | 2 |
|  |  | 6 | 1 |
|  |  | 7 | 1 |
|  |  | 8 | 1 |
|  |  | 11 | 2 |
|  | Changes in employment situation (n=3) | 3 | 1 |
|  |  | 8 | 2 |
|  | Occupational category (n=3) | 5 | 3 |
|  | International Socio-Economic Index of Occupational Status (n=2) | n.a. | 2 |
|  | Experiencing difficulties (such as unemployment. reduced business activity, etc.) due to COVID-19 pandemic (n=2) | 5 | 2 |
| **Deprivation (n=2)** | Deprivation status (n=2) by Index of multiple deprivation (IMD) | Continuous variable | 2 |

n.a. = not applicable
