# Supplementary material for: Temporal dynamics of socioeconomic inequalities in depressive and anxiety symptoms during the COVID-19 pandemic: a scoping review
Source: Front Public Health. 2024 Jul 3;12:1397392. doi: 10.3389/fpubh.2024.1397392 (PMC11252079; doi:10.3389/fpubh.2024.1397392)
Supplement: Supplementary file 4 [file Data_Sheet_4.docx]

**Additional file 4**

Outcome measures

**Table:** Outcome measures of the included studies.

| **Outcome** | **Instrument** | **Number of studies** |
| --- | --- | --- |
| **Depression**  **(n=38)** | Patient Health Questionnaire – 9 (PHQ-9) | 16 |
|  | Patient Health Questionnaire – 2 (PHQ-2) | 8 |
|  | Depression Anxiety Stress Scale – 21 (DASS-21) depression subscale | 3 |
|  | Patient Health Questionnaire – 8 (PHQ-8) | 2 |
|  | One question “Have you ever felt depressed or despaired that might interfere with daily life in a recent year?” | 2 |
|  | Beck's Depression Inventory (BDI) | 1 |
|  | Patient Health Questionnaire – 9 (PHQ-9) and Short Mood and Feelings Questionnaire (SMF-Q) | 1 |
|  | Major Depressive Episodes (MDEs) Questionnaire | 1 |
|  | Zung Self-Rating Depression Scale | 1 |
|  | Brief Symptom Inventory (BSI) Scale | 1 |
|  | Center for Epidemiologic Studies Depression (CES-D) Scale | 1 |
|  | Mini International Neuropsychiatric Interview (MINI) | 1 |
| **Anxiety**  **(n=24)** | Generalized Anxiety Disorder Scale– 2 (GAD-2) | 8 |
|  | Generalized Anxiety Disorder Scale– 7 (GAD-7) | 7 |
|  | Depression Anxiety Stress Scale – 21 (DASS-21) anxiety subscale | 2 |
|  | Institute for Personality& Ability Testing (IPAT) Anxiety Scale | 1 |
|  | State-Trait Anyiety Inventory – State subscale | 1 |
|  | Mini International Neuropsychiatric Interview (MINI) | 1 |
|  | Mini – Social Phobia Inventory (Mini-SPIN) | 1 |
|  | Overall Anxiety Severity and Impairment Scale (OASIS) | 1 |
|  | Generalized Anxiety Disorder Scale– 7 (GAD-7) and Short Warwick Edinburgh Mental Wellbeing Scale | 1 |
|  | Brief Symptom Inventory (BSI) Scale | 1 |
| **Depression & Anxiety combined (n=11)** | Patient Health Questionnaire – 4 (PHQ-4) | 6 |
|  | Mini International Neuropsychiatric Interview (MINI) | 1 |
|  | Mental Health Inventory – 5 (MHI-5) and Short Form – 36 (SF-36) | 1 |
|  | Mental Health Inventory – 5 (MHI-5) | 1 |
|  | Patient Health Questionnaire – 2 (PHQ-2), Generalized Anxiety Disorder Scale– 7 (GAD-7), Patient-Reported Outcome Measures Information System (PROMIS) anxiety | 1 |
|  | EuroQol- 5 Dimensions- 5 Levels (EQ-5D-5L) | 1 |
