# Supplementary material for: Temporal dynamics of socioeconomic inequalities in depressive and anxiety symptoms during the COVID-19 pandemic: a scoping review
Source: Front Public Health. 2024 Jul 3;12:1397392. doi: 10.3389/fpubh.2024.1397392 (PMC11252079; doi:10.3389/fpubh.2024.1397392)
Supplement: Supplementary file 5 [file Data_Sheet_5.docx]

**Additional file 5**

Observation periods


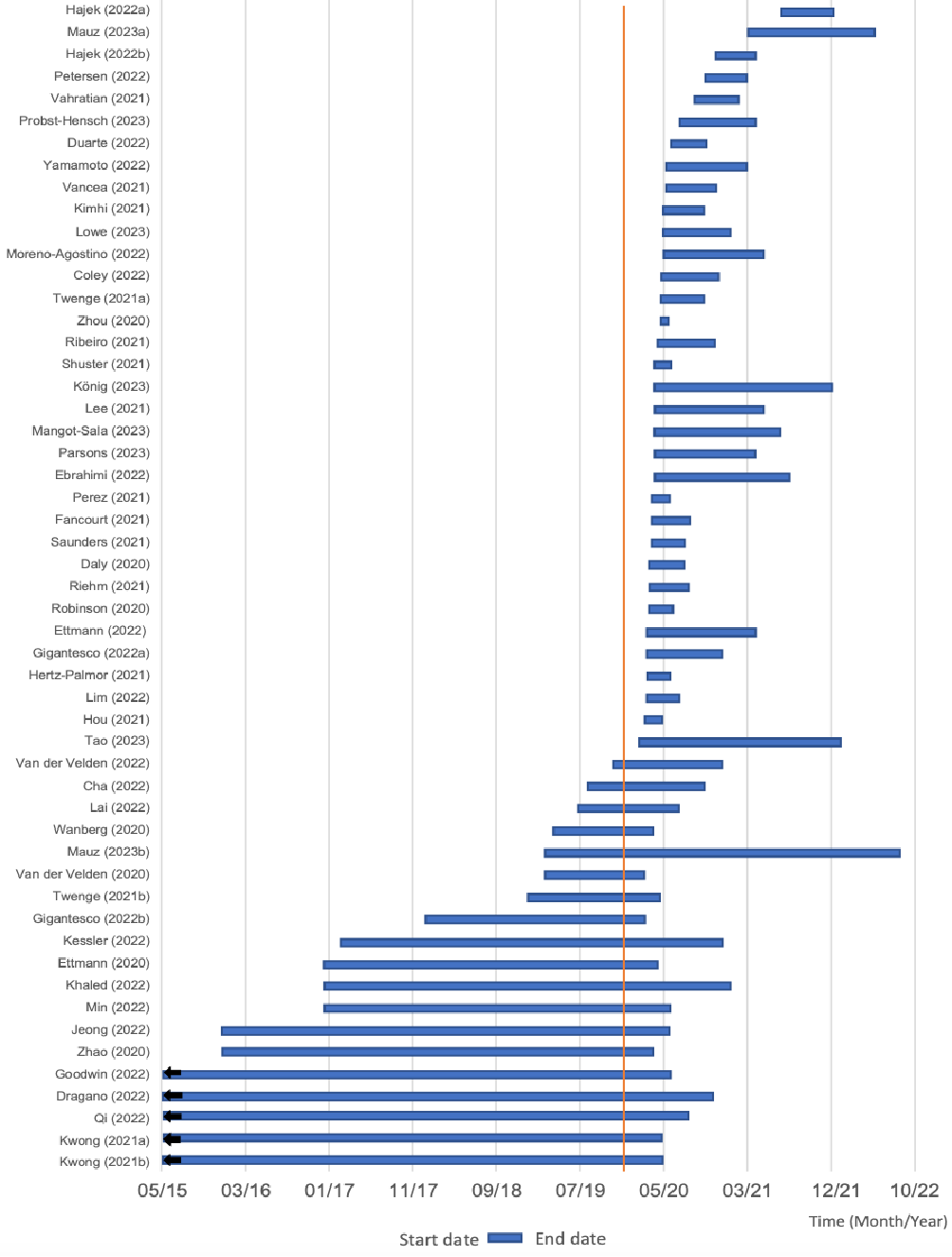


**Figure:** Observation periods of the included studies. The red line indicates the start of COVID-19 pandemic (11th March 2020). Four studies are shown twice in the figure leading to a total number of 53 bars (see also table 2 in the manuscript): Kwong et al. (2021) used data from two different cohorts. Mauz et al. (2023) present results regarding depressive symptoms before to during the pandemic as well as anxiety measures during the pandemic. Gigantesco et al., (2022) and Twenge et al. (2022) compared measures of anxiety and depressive symptoms before to during the pandemic as well as during the pandemic.

The graph shown is intended to give a broad overview of the periods of the studies examined. The studies are ordered by the starting point of the analyses with the latest starting point shown first. For presentation reasons, only the first author and the year of publication are shown. Thus, all studies starting to the left of the red line include analyses before to during the pandemic, while the studies to the right are during the pandemic. If there is a letter after the year of publication, the study either had different analyses about the time frame, from before to during the pandemic or within the pandemic (48-50), or the study covered different periods for the survey of depression and anxiety disorders. . Hajek 2022a and 2022b represent two studies outlined in the bibliography and Table 3 (56, 57). The start of the survey periods of the five lowest studies lays outside the figure’s range of display.
